# Simultaneous versus staged bilateral total hip arthroplasty: a systematic review and meta-analysis

**DOI:** 10.1186/s13018-022-03281-4

**Published:** 2022-08-13

**Authors:** Akam Ramezani, Amirhossein Ghaseminejad Raeini, Amirmohammad Sharafi, Mehrdad Sheikhvatan, Seyed Mohammad Javad Mortazavi, Seyyed Hossein Shafiei

**Affiliations:** 1 Orthopedic Department, Orthopedic Surgery Research Center (OSRC), Sina University Hospital, Tehran University of Medical Sciences, Tehran, Iran; 2 Heidelberg Medical Hospital, Heidelberg, Germany; 3 Joint Reconstruction Research Center (JRRC), Tehran University of Medical Sciences, Tehran, Iran

**Keywords:** Total hip arthroplasty, Meta-analysis, Bilateral total hip replacement, Cost–benefit analysis, Complications, Functional outcomes

## Abstract

**Background:**

Total hip arthroplasty is a common orthopedic surgery for treating primary or secondary hip osteoarthritis. Bilateral total hip replacement could be performed in a single stage or two separate stages. Each surgical procedure's reliability, safety, and complications have been reported controversially. This study aimed to review the current evidence regarding the outcomes of simultaneous and staged bilateral total hip arthroplasty.

**Methods:**

We conducted a meta-analysis using MEDLINE, EMBASE, Web of Science, and Scopus databases. Eligible studies compared complications and related outcomes between simultaneous and staged bilateral THA. Two reviewers independently screened initial search results, assessed methodological quality, and extracted data. We used the Mantel–Haenszel method to perform the meta-analysis.

**Results:**

In our study, we included 29,551 patients undergoing simBTHA and 74,600 patients undergoing stgBTHA. In favor of the simBTHA, a significant reduction in deep vein thrombosis (DVT) and systemic, local, and pulmonary complications was documented. However, we evidenced an increased pulmonary embolism (PE) and periprosthetic fracture risk in simBTHA. In the simBTHA, total blood loss, length of hospital stay, and total cost were lower.

**Conclusion:**

This meta-analysis shows that simultaneous bilateral THA accompanies fewer complications and lower total cost. Well-designed randomized controlled trials are needed to provide robust evidence.

**Supplementary Information:**

The online version contains supplementary material available at 10.1186/s13018-022-03281-4.

## Background

Total hip arthroplasty (THA) is one of the most common orthopedics surgeries. It is the preferred cost-effective treatment for osteoarthritis and other end-stage hip abnormalities. Patients experience a significant improvement in joint function as well as the quality of life following THA [1]. Studies suggest a rising trend in the number of performed THAs during the last decade [2]. From 2000 to 2014, the number of annual performed THAs increased by 105% in the USA. It is also projected that by 2030, this number will increase by 71.2%, reaching 635,000 procedures per year [3]. Total hip replacement also imposes a high economic burden on healthcare systems, with US hospitals bearing a staggering cost of $ 15 billion annually [4].

Patients scheduled for bilateral THA usually undergo two different timing sets of surgeries: simultaneous or staged. Simultaneous BTHA is performed in single hospital admission and under the same anesthesia. On the other hand, staged BTHA is executed at separate intervals in two hospitalizations and under two distinct anesthesia [5]. In 1971, Charnley et al. introduced simultaneous THA for bilateral hip pathologies, a noteworthy revolution in orthopedic science [5, 6]. Since then, there has always been controversy over which method could have better outcomes.

In 2016, Shao et al. conducted a systematic review comparing simBTHA and stgBTHA. It was revealed that surgery time, deep vein thrombosis (DVT), and major systemic complications were significantly lower in simBTHA compared to stgBTHA [7]. In 2019, another systematic investigation performed by Huang et al. also demonstrated lower rates of DVT, pulmonary embolism (PE), and respiratory complications in simBTHA [8].

There is still debate concerning this critical issue, and many original studies have been conducted since the last published systematic review. Previous reviews have focused on systemic and surgical complications, blood loss, operation time, and mortality as their primary outcomes. Essential factors such as readmission, revision, hip joint function, and cost have been considered less. Thus, a thorough review of the available data is required to identify the best way to perform bilateral THAs. The forthcoming systematic review aims to make a more comprehensive and accurate comparison between simultaneous and staged BTHA with a higher sample size and additional related outcomes.

## Method

The protocol of this study was registered on PROSPERO (CRD42022310240). We followed the Cochrane guidelines for meta-analysis during the process [9]. Our study phases were based on the Preferred Reporting Items for Systematic Reviews and Meta-Analysis (PRISMA) guidelines [10]. The PRISMA checklist is presented in Additional file 1.

### Search strategy

We searched the electronic databases MEDLINE, Web of Science (WOS), Embase, and Scopus for relevant articles in any published language; the last updating search was performed on February 15, 2022. The keywords are exhibited in Additional file 2. In addition, we explored the reference part of the articles that fulfilled our eligibility criteria. We also used the “related articles” feature in PubMed to avoid probable missing.

### Eligibility criteria

PICOS categories (population, intervention, comparator, outcomes, and study design) were applied to define our inclusion criteria. We included studies only if they were executed to compare mortality, complications, costs, or other possible outcomes between simBTHA and stgBTHA. Eligible study designs were randomized controlled trials (RCTs), non-randomized clinical trials, prospective and retrospective cohort studies, and case–control investigations. We did not impose any restrictions on the length of follow-up and year of publication. Exclusion criteria were reviews, research letters, conference abstracts, non-English articles, duplicate publications, irrelevant articles, non-human models, studies comparing simBTHA to unilateral THA, and resurfacing or revision surgery.

Systemic complications were defined as cardiovascular, pulmonary, gastrointestinal, urologic, and neurologic complications, hypotension, anemia, DVT, and PE. Notably, we did not include PE in the pulmonary complications in the meantime of analysis. Local complications in our study were defined as wound infection, decubitus ulcer, hematoma, dehiscence, neurapraxia, vascular injury, accidental laceration or puncture, chronic soft tissue pain, neuroma, wound drainage, superficial infection, and ectopic ossification.

### Data extraction

We imported all the studies into Rayyan online tool [11] in order to screen conveniently. After resolving duplicates, two researchers (AR, AS) completed an initial independent review to determine if the studies met the inclusion criteria hinged upon the title and abstract. Then, the two prior reviewers (AR, AS) evaluated each in the full-text screening phase. In case of any discrepancy, a third reviewer (AG) became involved and resolved it.

We prepared an electronic spreadsheet according to the Cochrane's template for data extraction of intervention reviews. Two separate reviewers fulfilled the data extraction (AR, AG). We acquired the following data from the studies: first author's name, publication year, country, study design, the sample size, mean age, gender, mean body mass index (BMI), American Society of Anesthesiology (ASA) classification, the interval between stages, duration of follow-up, primary and secondary outcomes including mortality, DVT, PE, fracture, dislocation, deep infection, any other complications, revision, readmission, operation time, blood loss, blood transfusion, length of hospital stay (LOS), hospital cost, and functional measures. Raw data were reviewed by another researcher (AS) to settle any disagreement. We also tried to contact the corresponding authors of the included articles regarding raw data or missing information. Patients with an ASA score of 1 or 2 were categorized as ‘low risk,’ and patients with an ASA score of 3 or 4 were categorized as ‘high risk’ [12].

### Methodology assessment

To assess the quality of each study, we employed the Newcastle–Ottawa Scale (NOS) for observational and non-randomized investigations. Briefly, the NOS evaluates a study according to three main characteristics: selection of groups, comparability, and outcome assessment [13]. We judged the quality of included studies according to the previous classification described in a meta-analysis by Simunovic et al. [14]. Studies with a score > 6 were categorized as high quality. Those with a score of 5 or 6 were classified as medium quality. Articles scored less than 5 were assigned as a low-quality study. Concerning randomized clinical trials (RCTs), we utilized the Cochrane Collaboration tool to assess the risk of bias. Two reviewers (AR, AS) independently assessed each study's quality. Disagreements were determined by consensus or involvement of the corresponding author (SHS).

### Statistical analysis

We performed meta-analysis using the Comprehensive Meta-Analysis software (Biostat, Englewood, NJ, USA, Version 3.3) if three or more studies reported a particular outcome. For dichotomous variables, odds ratios (ORs) were calculated and pooled for all investigations. Meta-analysis of dichotomous variables was committed through the Mantel–Haenszel (MH) method, with 95% confidence intervals (CI). Meta-analysis of continuous data was performed by applying the mean and standard deviation of outcome measures with 95% confidence intervals (CI). For studies that reported only data ranges without standard deviations, we calculated SDs using the formula suggested by Walter & Yao [15]. A *p* value less than 0.05 was considered statistically significant. We analyzed heterogeneity among the studies using the *I*^2^ test [16]. *I*^2^ > 50% with a *p* value < 0.05 suggested high heterogeneity. A fixed-effects model was utilized if low statistical heterogeneity among the studies was discovered (*I*^2^ < 50%). A random-effects model was used if high heterogeneity became proven. We also detected potential publication bias by using Begg’s funnel plots and the Egger test [17].

## Results

### Search results

**Fig. 1:**
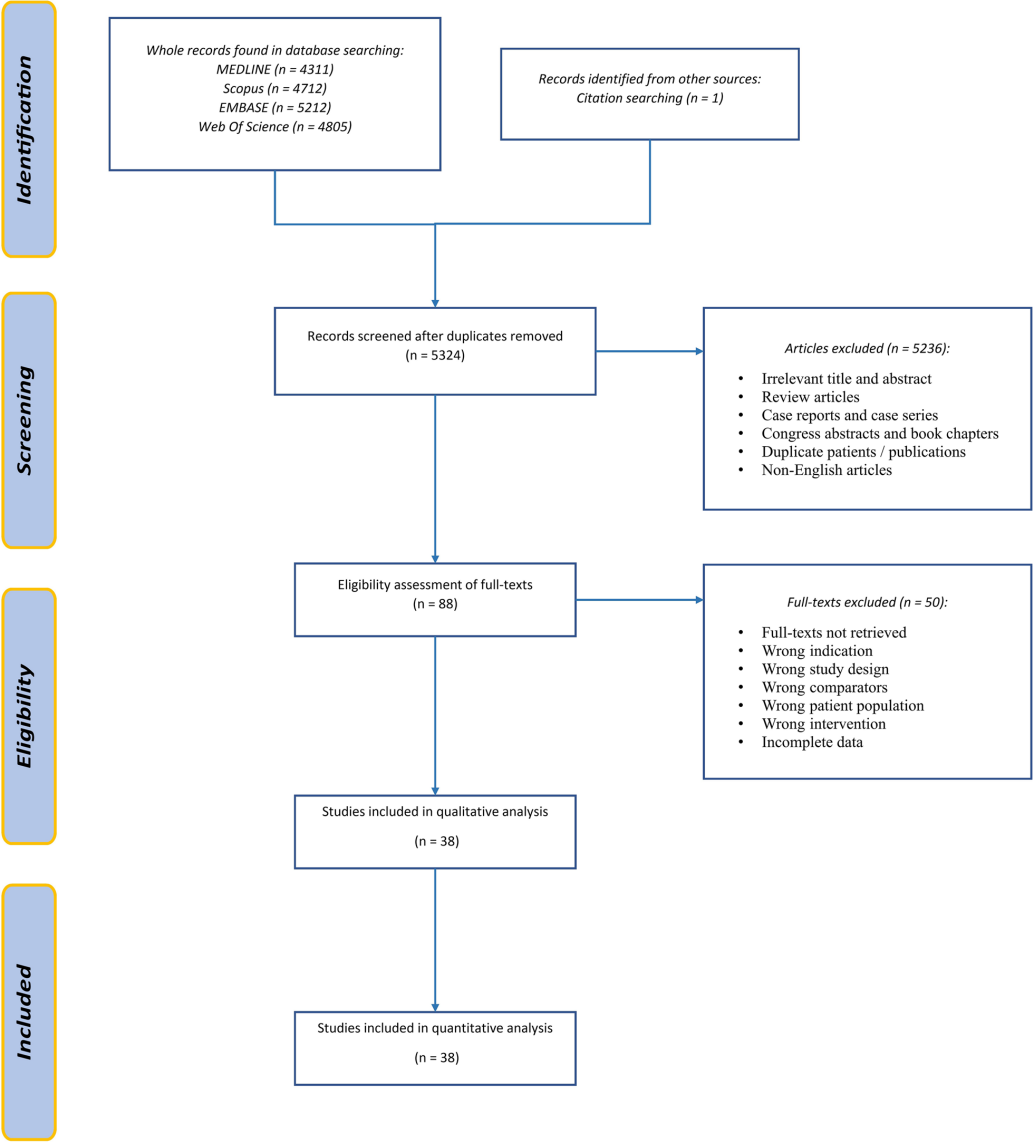
PRISMA flowchart showing identification, screening, and inclusion of studies for review

After deleting duplications, we identified 5324 potentially relevant titles from the mentioned databases. Based on the titles and abstracts, 5236 publications were excluded. Full texts of 88 remaining publications were screened. Finally, in this systematic review, 38 studies, including 104,151 patients (29,551 simBTHA and 74,600 stgBTHA), were entered into the quantitative analysis. A flowchart summarizing the selection process is provided in Fig. 1.

### Study characteristics

Among the 38 included studies, 2 studies [18, 19], including 348 patients, were RCTs and 36 studies were non-RCTs [20–55]. The baseline characteristics of the articles are displayed in Table 1. Studies were in the English language and were published from 1978 to 2022. The duration of follow-up was at least 3 months. The sample size of included studies ranged from 15 to 42,238. The mean age of participants was 57.6 years for simBTHA and 63.2 years for stgBTHA. The male-to-female ratio was 1:1.29. Raw data for ASA classification were reported in 14 studies [18, 19, 24, 25, 33–35, 37, 41, 42, 45–47, 49]. Regarding ASA score, 13% and 18% of patients in simBTHA and stgBTHA were considered high risk (ASA 3 or 4), respectively (Table 1).

**Table 1:** Main baseline characteristics of the included studies

Author (year)	Country	Study design	Simultaneous bilateral THA					Staged bilateral THA						Mean follow-up (range)
			n	Age (mean, year)	Gender (male/female)	BMI (mean. Kg/m^2^)	ASA (1/2/3/4) (percentage)	n	Age (mean, years)	Gender (male/female)	BMI (mean. Kg/m^2^)	ASA (1/2/3/4) (percentage)	Time interval between stages	
Agarwal et al. (2016)	India	Retrospective cohort	48	52	20:28	–	–	56	54	26:30	–	–	4.2 days	70 (36–82 months)
Aghayev et al. (2010)	Switzerland	Registry	247	59	116:131	–	–	1572	62.5	786:786	–	–	–	60 months
Alfaro-Adrián et al. (1999)	Brazil	Retrospective cohort	95	65	40:55	–	43/37/19/1	107	63.9	42:65	–	60/29/8/3	10.1 months	–
Berend et al. (2007)	USA	Retrospective cohort	167	52.7	100:67	29.7	–	110	57.3	47:63	30.8	–	8.1 months	28.5 months
Bhan et al. (2006)	India	Randomized clinical trial	83	46.6	54:29	–	59/31/10/0	85	43.4	51:34	–	64/29/6/1	3–7 months	60 months
Brown et al. (2017)	USA	Retrospective cohort	15	56.9	8:7	26.4	Mean = 1.8 ± 0.6	44	60.2	24:20	27.8	Mean = 2.2 ± 0.6	0.90 ± 0.89 years	3 months
Calabro et al. (2020)	Australia	Registry	2779	–	6214:6145**	–	19/60/20/1	9580	–	6214:6145**	–	10/58/30/2	–	Minimum = 1.5 years
Eggli et al. (1995)	Switzerland	Prospective cohort	64	54	133:122**	–	–	191	61	133:122**	–	–	96 days	1.5 years
Garland et al. (2015)	Sweden	Registry	1680	–	767:913	26.9	33/54/13/0	40,558	–	16,356:24,202	27.4	23/61/16/0	–	to the day of death
Goh et al. (2022)	USA	Retrospective cohort	220	60.8	341:330^Δ^	30	–	170	64	123:210^Δ^	30.7	–	–	3 months
Guo et al. (2020)	China	Retrospective cohort	863	49	604:259	24.7	31/68/1/0	282	52.5	152:130	24.8	27/69/4/0	–	Minimum = 3 months
Hooper et al. (2009)	New Zealand	Registry	303	61	–	–	–	743	61	–	–	–	–	6 months
Hou et al. (2021)	China	Retrospective case control	100	54	30:70	24.6	–	100	57	29:71	24.6	–	–	–
Houdek et al. (2017)	USA	Retrospective case control	94	52.2	54:40	27.1	7/78/15/0	94	52.1	54:40	27.8	7/80/13/0	3 months	48 months
Inoue et al. (2021)	USA	Retrospective cohort	256	58.2	155:101	27.8	–	387	62.5	176:211	28.4	–	31.5 months	90 days
Johnston et al. (2011)	Scotland	Retrospective cohort	68	61.5	26:42	27.4	–	526	66.5	208:318	27.2	–	–	1.5 years (24–108 months)
Kamath et al. (2016)	Switzerland	Retrospective cohort	41	60.7	24:17	–	18/58/24/0	44	68.7	18:26	–	12/65/23/0	–	Minimum = 24 months
Kim et al. (2017)	South Korea	Retrospective cohort	63	43.1	39:24	22.9	30/60/10/0	60	43.5	32:28	23.3	30/66/4/0	4.8 months	60.2 months
Lindberg‑Larsen et al. (2013)	Denmark	Registry	103	55.7	59:44	–	–	577	66.9	234:343	–	–	–	Max = 415 days
Lorenze et al. (1998)	USA	Retrospective case control	40	–	20:20	–	–	40	–	20:20	–	–	–	–
Martin et al. (2016)	Canada	Retrospective case control	12	58.9	–	27.9	Mean = 2.2 ± 0.4	12	63.9	–	26.3	Mean = 2.2 ± 0.6	–	–
Mou et al. (2021)	China	Retrospective cohort	11	–	10:1	22.7	–	12	–	10:2	22.7	–	40.8 days	80.9 months
Panchal et al. (2021)	India	Retrospective case control	54	–	27:27	–	–	54	–	27:27	–	–	–	62.4 months
Partridge et al. (2019)	UK	Registry	2507	60.6	1178:1329	–	–	9915	65.5	3966:5949	–	–	–	3 months
Parvizi et al. (2006)	USA	Retrospective case control	98	53	53:45	28.8	11/72/17/0	98	65	46:52	30.2	1/51/48/0	138 days	Minimum = 6 months
Poultsides et al. (2017)	USA	Retrospective cohort	1946	56.3	1000:946	–	–	1839	63.1	746:1093	–	–	5–365 days	–
Quadri et al. (2015)	Pakistan	Retrospective cohort	34	39	30:4	25	29/57/7/7	14	42	6:8	27	14/57/29/0	–	–
Rasouli et al. (2014)	USA	National database	14,798	58.4	–	–	–	1532	60.3	–	–	–	–	–
Reuben et al. (1998)	USA	Retrospective case control	7	49	4:3	–	Mean = 2.5 ± 0.5	8	57	1:7	–	Mean = 1.7 ± 0.7	–	–
Saito et al. (2010)	Japan	Retrospective case control	49	59	6:43	23.5	–	40	61.9	4:36	23.8	–	30.7 days	5.5 years (24–120 months)
Salvati et al. (1978)	USA	Retrospective cohort	122	–	–	–	–	339	–	–	–	–	–	36 months
Schlegelmilch et al. (2017)	Canada	Retrospective case control	26	–	–	–	–	6	–	–	–	–	–	12 months
Seol et al. (2015)	Korea	Retrospective cohort	147	41.9	112:35	23.7	54/41/5/0	59	46.3	45:14	23.8	46/46/8/0	18.7 months	34.4 months
Shih et al. (1985)	China	Retrospective cohort	20	40.7	17:3	–	–	15	46.6	13:2	–	–	–	365–530 days
Taheriazam et al. (2019)	Iran	Randomized clinical trial	90	59.3	59:31	28.4	score 1 or 2	90	59.1	52:38	28.7	score 1 or 2	6–12 months	24 months
Tan et al. (2019)	China	Retrospective cohort	256	52	143:113	23.8	41/49/8/2	256	54.9	120:136	23.8	38/54/7/1	–	3 months
Triantafyllopoulos et al. (2016)	USA	Retrospective cohort	1808	56.3	930:878	–	–	4842	62.3	1995:2847	–	–	249–1710 days	112.6 months
Villa et al. (2019)	USA	Retrospective cohort	61	55.4	40:21	27.5	15/69/16/0	143	63.1	63:80	27.7	7/69/24/0	461	–

*THA* total hip arthroplasty, *n* number, *BMI* body mass index, *ASA* American Society of Anesthesiology

**This is a report of the gender in whole sample size (not reported in separated groups)

Δ This is a report of the gender in whole total joint arthroplasty sample size (not reported in separated groups; THA and TKA)

### Quality assessment

Randomization methods, outcome assessment blinding, incomplete outcome data, and selective data reporting were low risk for both RCTs. Although the allocation method was not reported in one RCT, all other included studies were observational, comprising one prospective cohort, seven registries, nineteen retrospective cohorts, and nine retrospective case controls. The risk-of-bias assessment results for both randomized and observational studies are summarized in Table 2.

**Table 2:** Quality assessment of the eligible studies

Author	Year	Study type	Random sequence generation	Allocation concealment	Blinding of participants and personnel	Blinding of outcome assessment	Incomplete outcome data	Selective reporting	Other bias
Bhan et al.	2006	Randomized clinical trial	Yes	Unclear	Unclear	Yes	Yes	Yes	No bias
Taheriazam et al.	2019	Randomized clinical trial	Yes	Yes	Unclear	Yes	Yes	Unclear	No bias

			Newcastle–Ottawa Scale (NOS)			
			Selection	Comparability	Exposure/Outcome	Total score
Agarwal et al.	2016	Retrospective cohort	3	1	2	6
Aghayev et al.	2010	Registry	3	1	2	6
Alfaro-Adrián et al.	1999	Retrospective cohort	3	1	2	6
Berend et al.	2007	Retrospective cohort	3	1	2	6
Brown et al.	2017	Retrospective cohort	3	2	1	6
Calabro et al.	2020	Registry	3	2	2	7
Eggli et al.	1995	Prospective cohort	3	2	2	7
Garland et al.	2015	Registry	3	1	2	6
Goh et al.	2022	Retrospective cohort	3	1	1	5
Guo et al.	2020	Retrospective cohort	3	1	2	6
Hooper et al.	2009	Registry	3	1	2	6
Hou et al.	2021	Retrospective case control	3	1	2	6
Houdek et al.	2017	Retrospective case control	3	1	2	6
Inoue et al.	2021	Retrospective cohort	3	1	1	5
Johnston et al.	2011	Retrospective cohort	4	1	2	7
Kamath et al.	2016	Retrospective cohort	4	2	2	8
Kim et al.	2017	Retrospective cohort	3	2	2	7
Lindberg‑Larsen et al.	2013	Registry	4	1	2	7
Lorenze et al.	1998	Retrospective case control	3	1	1	5
Martin et al.	2016	Retrospective case control	4	1	0	5
Mou et al.	2021	Retrospective cohort	4	1	2	7
Panchal et al.	2021	Retrospective case control	3	2	2	7
Partridge et al.	2019	Registry	3	2	2	7
Parvizi et al.	2006	Retrospective case control	3	1	2	6
Poultsides et al.	2017	Retrospective cohort	3	2	1	6
Quadri et al.	2015	Retrospective cohort	3	2	2	7
Rasouli et al.	2014	National database	3	1	2	6
Reuben et al.	1998	Retrospective case control	3	1	1	5
Saito et al.	2010	Retrospective case control	4	1	2	7
Salvati et al.	1978	Retrospective cohort	3	1	1	5
Schlegelmilch et al.	2017	Retrospective case control	3	1	1	5
Seol et al.	2015	Retrospective cohort	3	1	1	5
Shih et al.	1985	Retrospective cohort	2	1	2	5
Tan et al.	2019	Retrospective cohort	3	2	1	6
Triantafyllopoulos et al.	2016	Retrospective cohort	3	1	2	6
Villa et al.	2019	Retrospective cohort	4	1	2	7

### Mortality and complications

**Fig. 2:**
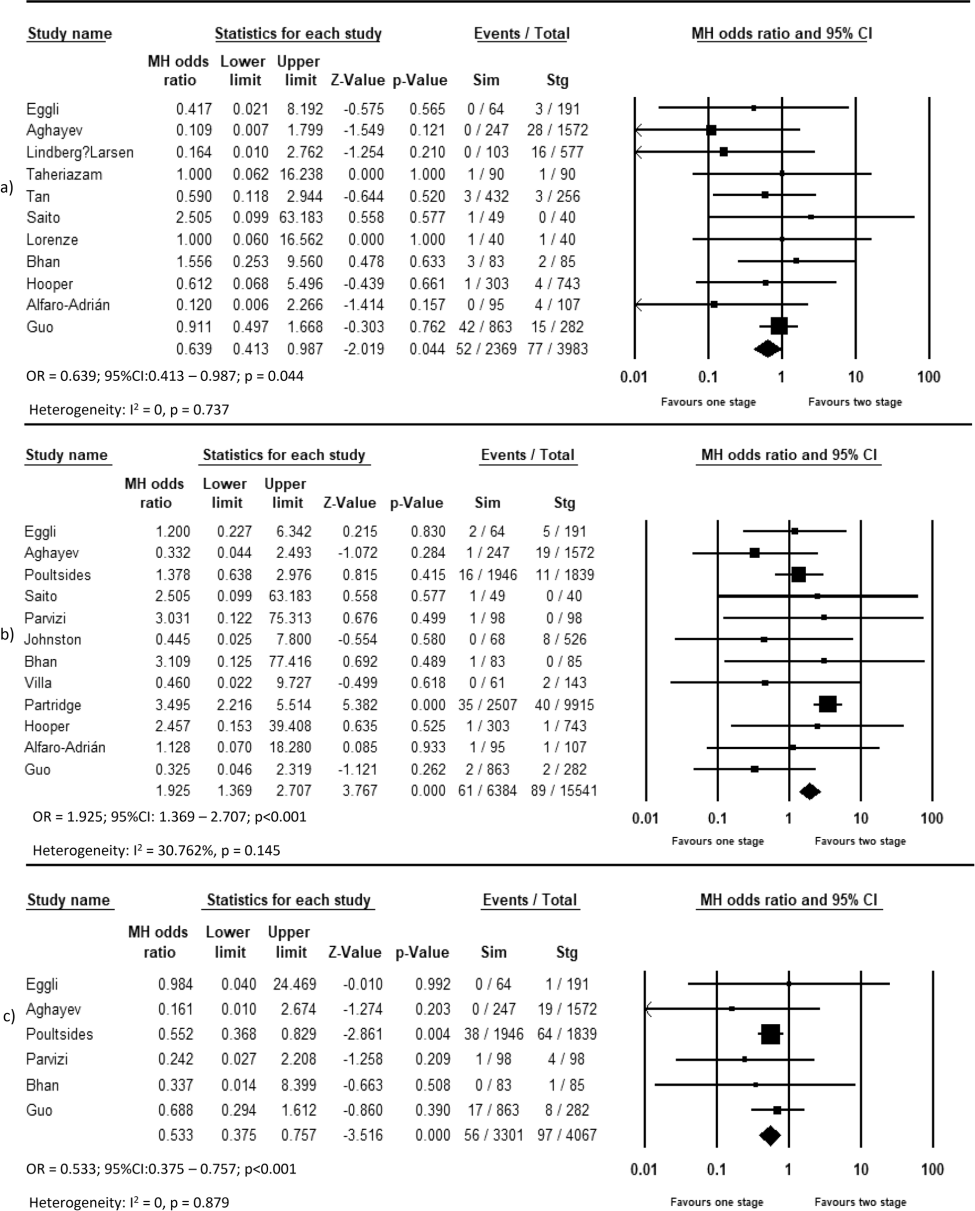
Forest plot of **a** DVT, **b** PE, and **c** pulmonary complications. M-H, Mantel–Haenszel; OR, odds ratio; 95% CI, 95% confidence interval

**Fig. 3:**
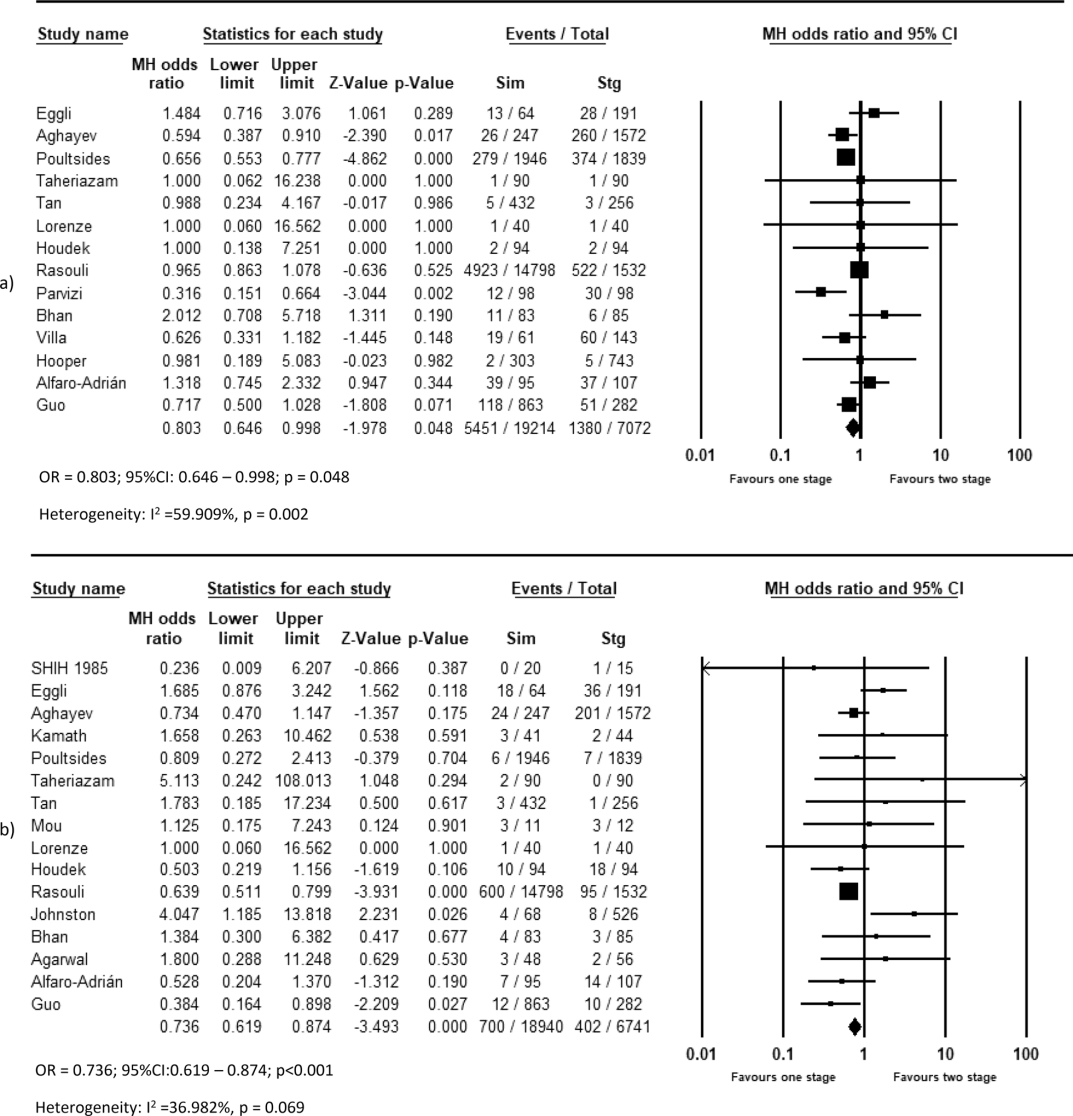
Forest plot of **a** systemic complications and **b** local complications. M-H, Mantel–Haenszel; OR, odds ratio; 95% CI, 95% confidence interval

**Fig. 4:**
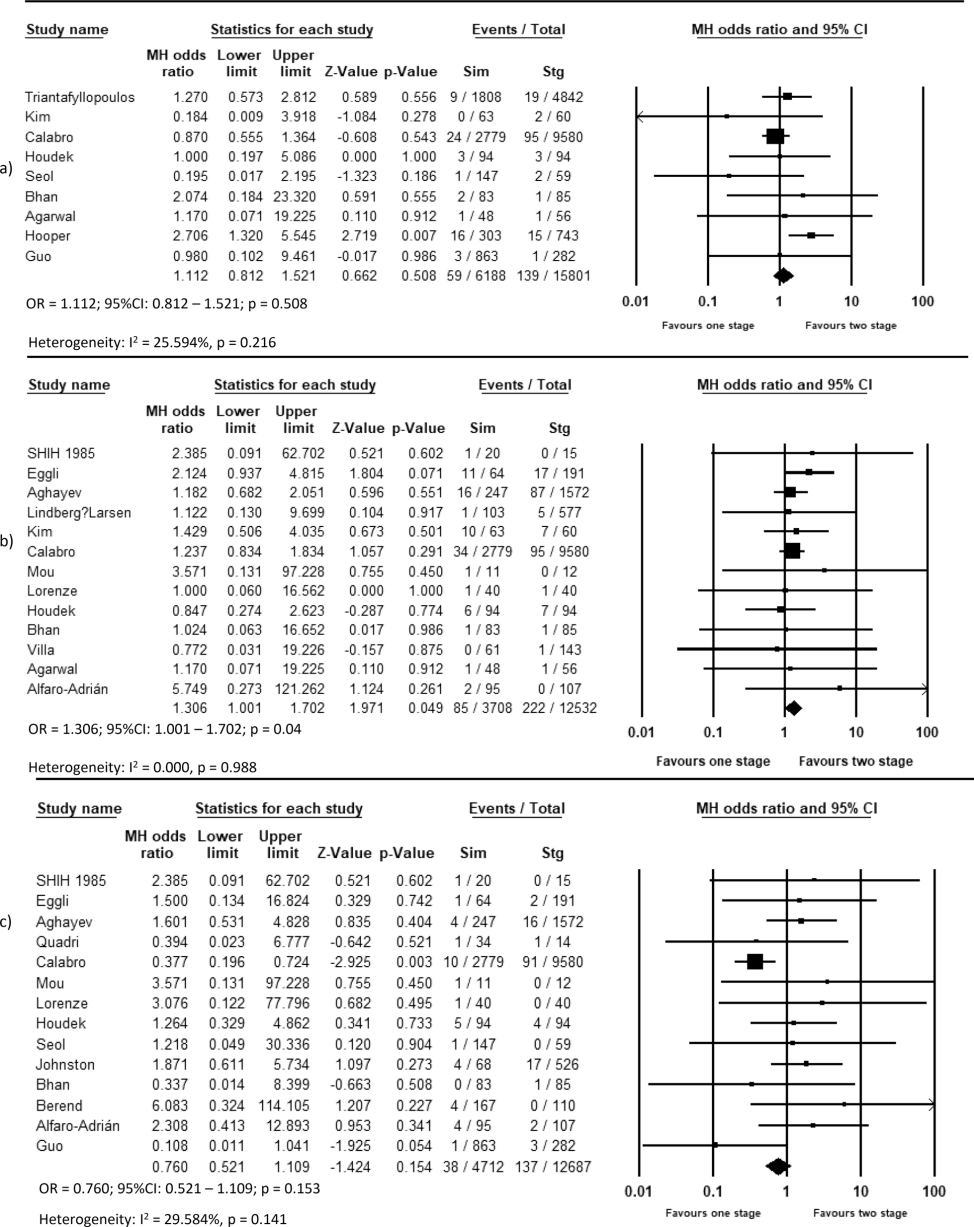
Forest plot of **a** periprosthetic joint infection, **b** periprosthetic fracture, and **c** dislocation. M-H, Mantel–Haenszel; OR, odds ratio; 95% CI, 95% confidence interval

**Fig. 5:**
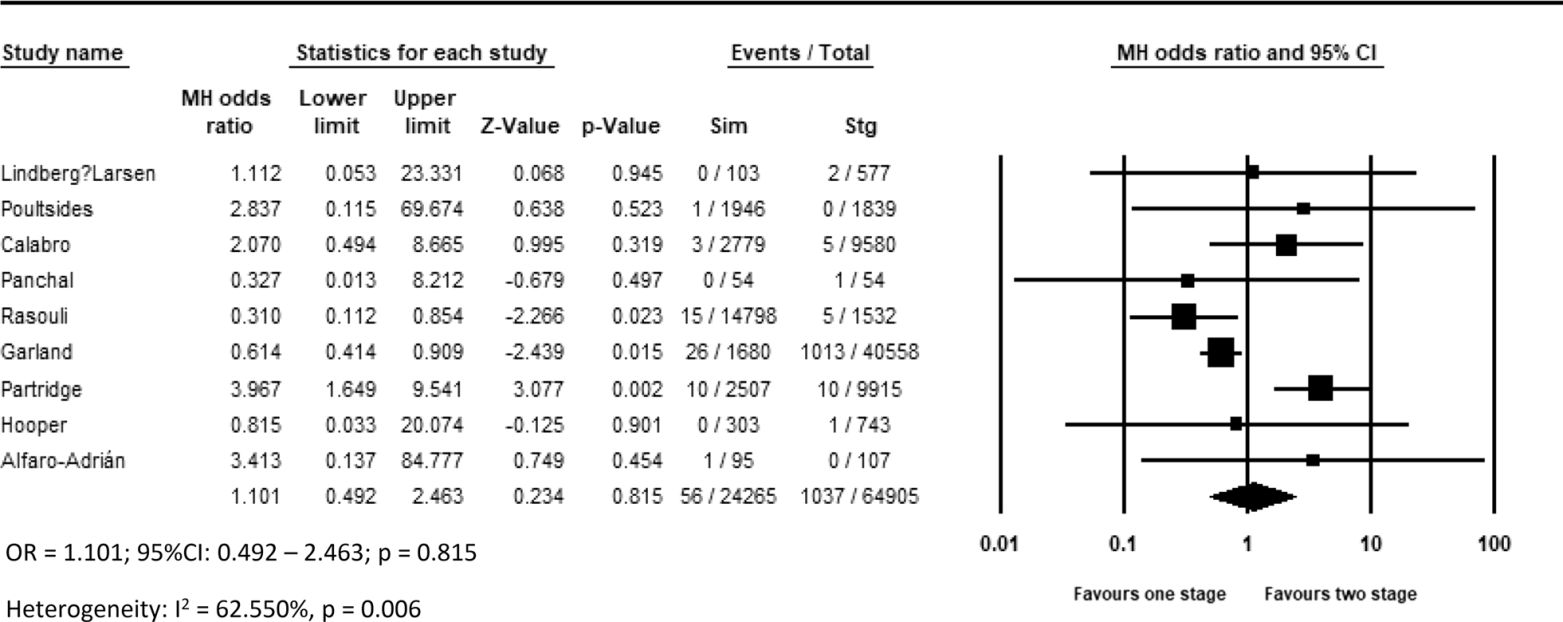
Forest plot of 90-day mortality. M-H, Mantel–Haenszel; OR, odds ratio; 95% CI, 95% confidence interval

Pooled analysis of 11 studies on DVT (OR = 0.639, *p* = 0.044, Fig. 2a), 12 studies on pulmonary complications (OR = 0.533, *p* < 0.001, Fig. 2c), 14 studies on systemic complications (OR = 0.803, *p* = 0.048, Fig. 3a), and 16 studies on local complications (OR = 0.736, *p* < 0.00, Fig. 3b) exhibited that these complications are lower in simBTHA. However, PE, reported in 12 studies (OR = 1.925, *p* < 0.001, Fig. 2b), and periprosthetic fracture, reported in 13 studies (OR = 1.306, *p* = 0.049, Fig. 4b), were higher in simBTHA. 90-day mortality, reported in eight studies (OR = 1.101, *p* = 0.815, Fig. 5), periprosthetic joint infection, reported in nine studies (OR = 1.112, *p* = 0.508, Fig. 4a), and dislocation, reported in 14 studies (OR = 0.760, *p* = 0.153, Fig. 4c), were similar between the two groups (Table 3).

**Table 3:** Summary of postoperative mortality and complications reported in each included study

Author	Year	Simultaneous bilateral THA									Staged bilateral THA								
		Mortality (n)	Deep infection (n)	Fracture (n)	Dislocation (n)	DVT (n)	PE (n)	Pulmonary complication (n)	Local complications (n)	Systemic complications (n)	Mortality (n)	Deep infection (n)	Fracture (n)	Dislocation (n)	DVT (n)	PE (n)	Pulmonary complication (n)	Local complications (n)	Systemic complications (n)
Agarwal et al.	2016	–	1	1	–	0	0	–	3	0	–	1	1	–	0	0	–	2	0
Aghayev et al.	2010	–	–	16	4	0	1	0	24	26	–	–	87	16	28	19	19	201	260
Alfaro-Adrián et al.	1999	1	–	2	4	0	1	–	7	39	0	–	0	2	4	1	–	14	37
Berend et al.	2007	–	–	–	4	–	–	–	–	–	–	–	–	0	–	–	–	–	–
Bhan et al.	2006	0	2	1	0	3	1	0	4	11	0	1	1	1	2	0	1	3	6
Brown et al.	2017	–	–	–	–	0	0	–	–	–	–	–	–	–	0	0	–	–	–
Calabro et al.	2020	3	24	34	10	–	–	–	–	–	5	95	95	91	–	–	–	–	–
Eggli et al.	1995	–	–	11	1	0	2	0	18	13	–	–	17	2	3	5	1	36	28
Garland et al.	2015	26	–	–	–	–	–	–	–	–	1013	–	–	–	–	–	–	–	–
Goh et al.	2022	–	–	–	–	–	–	–	–	–	–	–	–	–	–	–	–	–	–
Guo et al.	2020	–	3	–	1	42	2	17	12	118	–	1	–	3	15	2	8	10	51
Hooper et al.	2009	0	16	–	–	1	1	–	–	2	1	15	–	–	4	1	–	–	5
Hou et al.	2021	–	–	–	–	–	–	–	–	–	–	–	–	–	–	–	–	–	–
Houdek et al.	2017	0	3	6	5	–	–	–	10	2	0	3	7	4	–	–	–	18	2
Inoue et al.	2021	–	–	–	–	0	0	–	–	–	–	–	–	–	0	0	–	–	–
Johnston et al.	2011	–	–	–	4	0	0	–	4	–	–	–	–	17	0	8	–	8	–
Kamath et al.	2016	0	0	–	0	–	–	–	3	–	0	0	–	0	–	–	–	2	–
Kim et al.	2017	2	0	10	0	–	–	–	–	–	1	2	7	0	–	–	–	–	–
Lindberg‑Larsen et al.	2013	0	–	1	–	0	–	–	–	–	2	–	5	–	16	–	–	–	–
Lorenze et al.	1998	–	0	1	1	1	0	0	1	1	–	0	1	0	1	0	0	1	1
Martin et al.	2016	–	–	–	–	–	–	–	–	–	–	–	–	–	–	–	–	–	–
Mou et al.	2021	–	–	1	1	–	–	–	3	–	–	–	0	0	–	–	–	3	–
Panchal et al.	2021	0	0	–	0	0	–	–	–	–	1	0	–	0	0	–	–	–	–
Partridge et al.	2019	10	–	–	–	–	35	–	–	–	10	–	–	–	–	40	–	–	–
Parvizi et al.	2006	0	–	–	–	–	1	1	–	12	0	–	–	–	–	0	4	–	30
Poultsides et al.	2017	1	–	–	–	–	16	38	6	279	0	–	–	–	–	11	64	7	374
Quadri et al.	2015	–	–	–	1	–	–	–	–	–	–	–	–	1	–	–	–	–	–
Rasouli et al.	2014	15	–	–	–	–	–	–	600	4923	5	–	–	–	–	–	–	95	522
Reuben et al.	1998	–	–	–	–	–	–	–	–	–	–	–	–	–	–	–	–	–	–
Saito et al.	2010	0	0	–	–	1	1	–	–	–	0	0	–	–	0	0	–	–	–
Salvati et al.	1978	0	–	–	–	–	–	–	–	–	1	–	–	–	–	–	–	–	–
Schlegelmilch et al.	2017	–	–	–	–	–	–	–	–	–	–	–	–	–	–	–	–	–	–
Seol et al.	2015	0	1	–	1	–	0	–	–	0	0	2	–	0	–	0	–	–	0
Shih et al.	1985	0	–	1	1	0	–	–	0	0	0	–	0	0	0	–	–	1	0
Taheriazam et al.	2019	0	0	0	0	1	0	–	2	1	0	0	0	0	1	0	–	0	1
Tan et al.	2019	0	–	–	–	3	0	–	3	5	0	–	–	–	3	0	–	1	3
Triantafyllopoulos et al.	2016	–	9	–	–	–	–	–	–	0	–	19	–	–	–	–	–	–	0
Villa et al.	2019	–	1	0	–	0	0	–	3	19	–	1	1	–	0	2	–	2	60

*DVT* deep vein thrombosis, *PE* pulmonary embolism, *n* number, *THA* total hip arthroplasty

### Perioperative and postoperative relevant outcomes

**Fig. 6:**
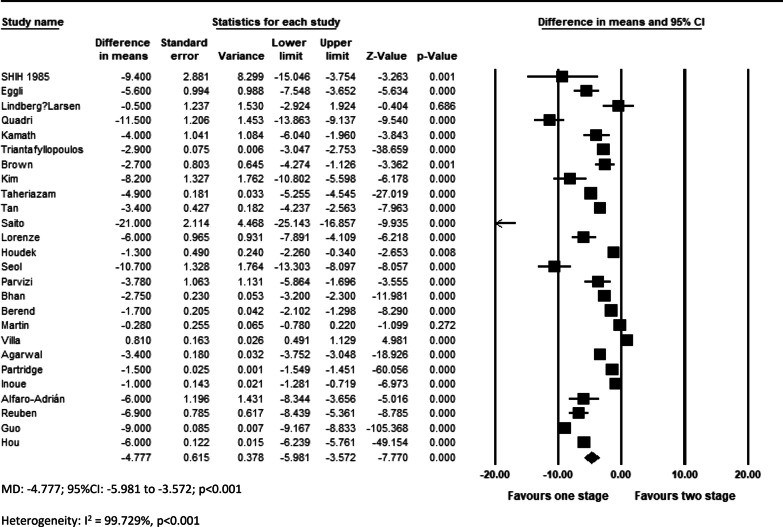
Forest plot of LOS. MD, mean difference; 95% CI, 95% confidence interval

**Fig. 7:**
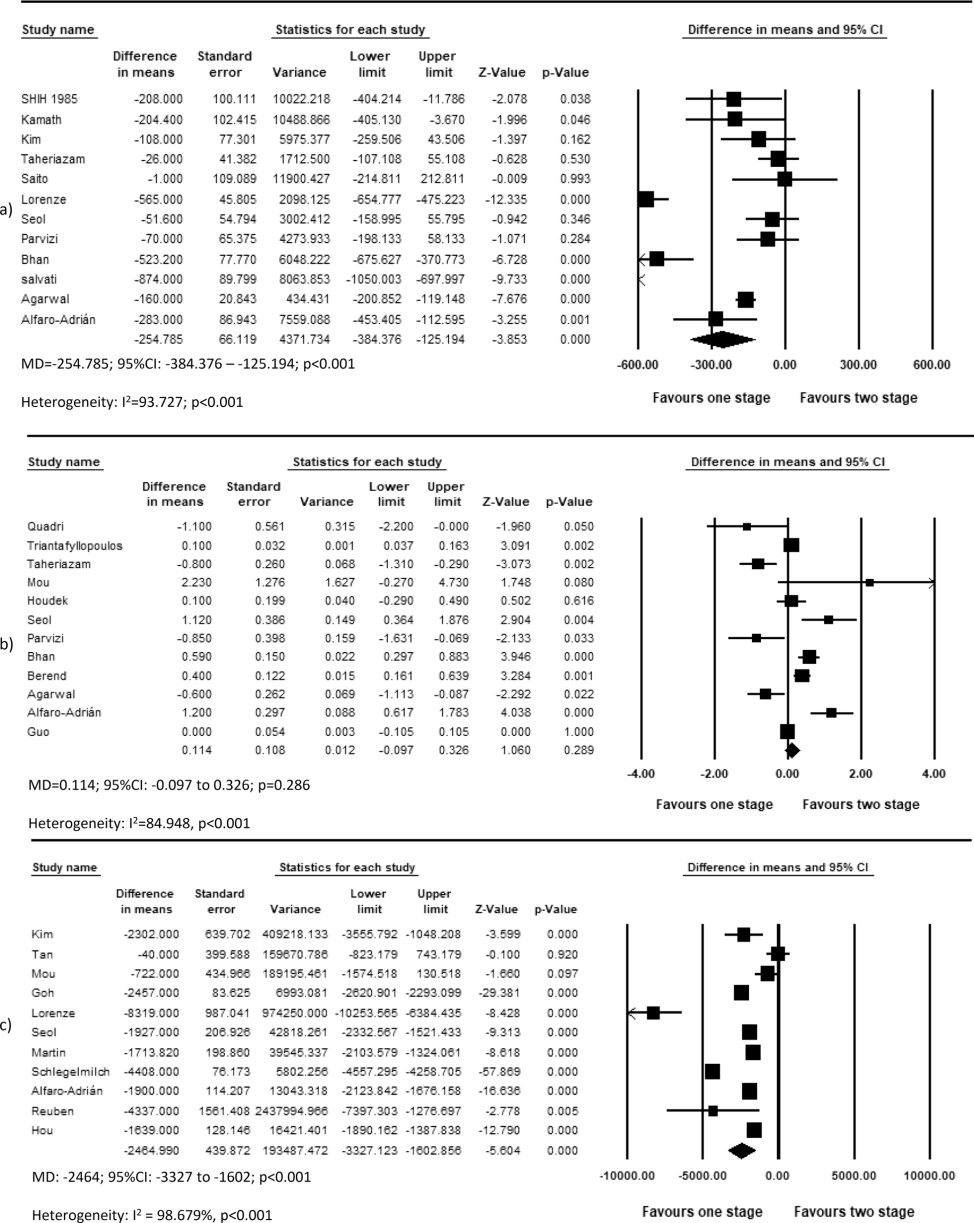
Forest plot of **a** total blood loss, **b** blood transfusion need, and **c** total cost. MD, mean difference; 95% CI, 95% confidence interval

**Fig. 8:**
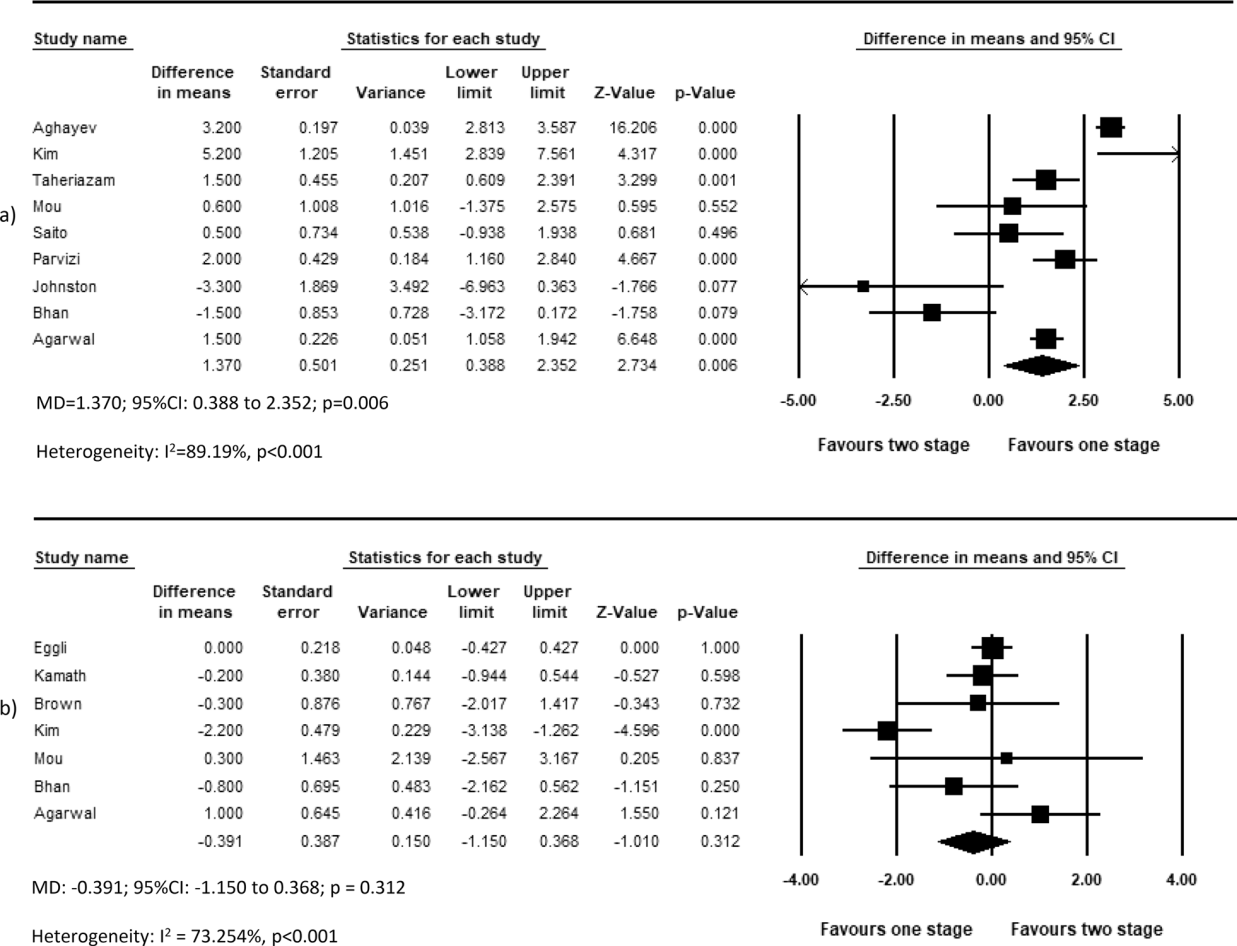
Forest plot of **a** postoperative HHS and **b** postoperative LLD. MD, mean difference; 95% CI, 95% confidence interval

**Fig. 9:**
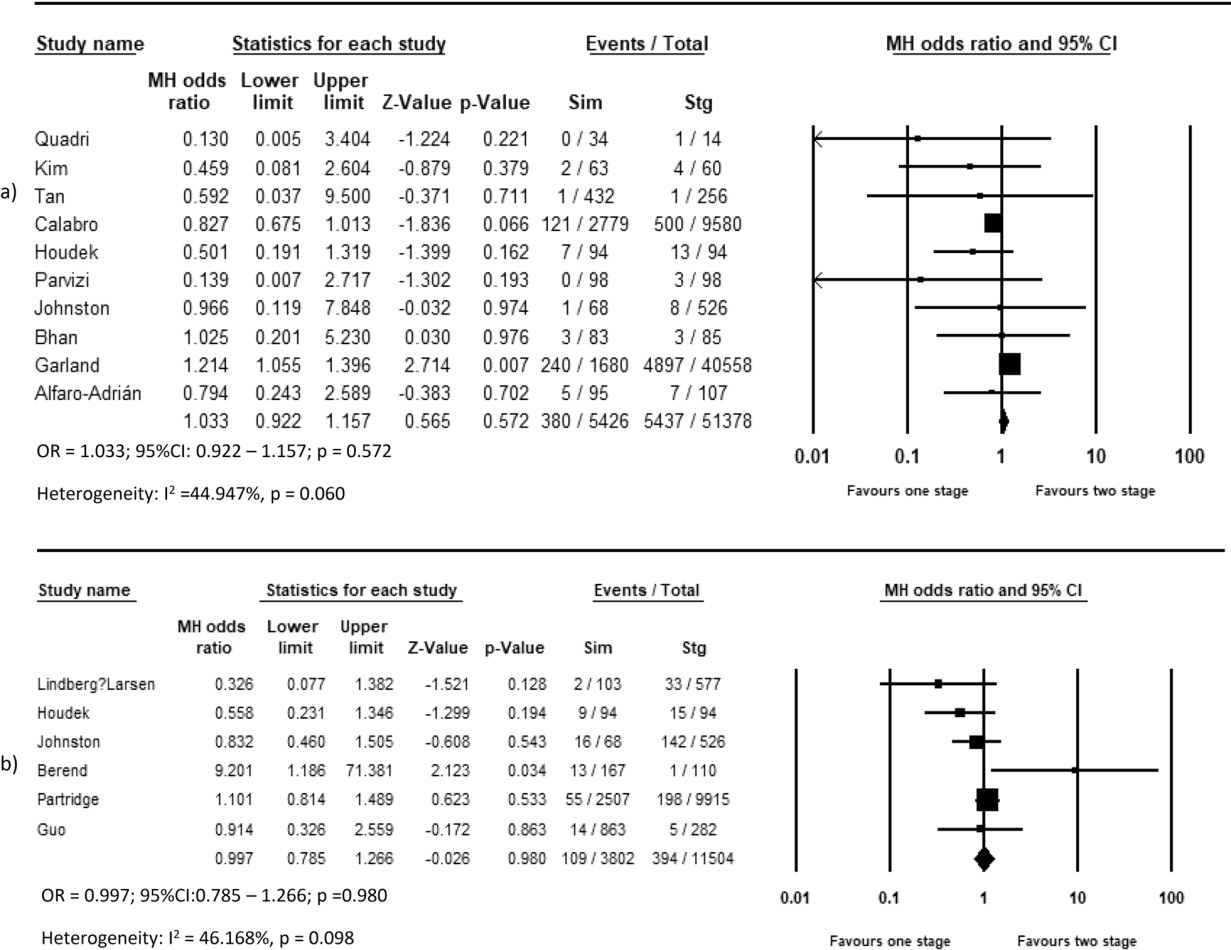
Forest plot of **a** revision and **b** readmission. MD, mean difference; 95% CI, 95% confidence interval

The overall effect of included studies demonstrated that simBTHA was lower in terms of length of stay (MD = −4.777, *p* < 0.001, Fig. 6) (26 studies), operation cost (USD) (MD = −2464, *p* < 0.001, Fig. 7c) (11 studies), and blood loss (MD = −254.785, *p* < 0.001, Fig. 7a) (12 studies). Pooled data of nine studies showed that the simBTHA group experiences a mean 1.37 point improvement over the stgBTHA group in postoperative Harris Hip Score (HHS) (MD = 1.370, *p* = 0.006, Fig. 8a). There was no significant difference in the revision rate (OR = 1.033, *p* = 0.572, Fig. 9a) (ten studies), readmission rate (OR = 0.997, *p* = 0.980, Fig. 9b) (six studies), blood transfusion rate (MD = 0.114, *p* = 0.286, Fig. 7b) (12 studies), and postoperative limb length discrepancy (LLD) (MD = −0.391, *p* = 0.312, Fig. 8b) (seven studies) (Tables 4 and 5).

**Table 4:** In-hospital important outcomes reported in each included study

Author	Year	Simultaneous bilateral THA					Staged bilateral THA				
		Operation time (min)	Hospital LOS (days)	Operation cost ($)	Transfusion (units)	Blood loss (ml)	Operation time (min)	Hospital LOS (days)	Operation cost ($)	Transfusion (units)	Blood loss (ml)
Agarwal et al.	2016	–	5.6 ± 0.8	–	1.6 ± 1.1	280 ± 86.7	–	9 ± 1.0	–	2.2 ± 1.5	440 ± 120.0
Aghayev et al.	2010	–	–	–	–	–	–	–	–	–	–
Alfaro-Adrián et al.	1999	202.6 ± 52.5	17 ± 9.0	9300 ± 750.0	3.9 ± 2.0	1579 ± 590.3	205.9 ± 41.3	23 ± 8.0	11,200 ± 860.0	2.7 ± 2.2	1862 ± 639.3
Berend et al.	2007	–	3.9 ± 1.5	–	0.8 ± 1.1	–	–	5.6 ± 1.9	–	0.4 ± 0.8	–
Bhan et al.	2006	207.42 ± 37.8	7.3 ± 1.3	–	2.4 ± 0.8	1473.9 ± 517.1	215.6 ± 37.4	10 ± 1.7	–	1.8 ± 1.1	1997.1 ± 490.8
Brown et al.	2017	–	5 ± 2.3	–	–	–	–	7.7 ± 2.8	–	–	–
Calabro et al.	2020	–	–	–	–	–	–	–	–	–	–
Eggli et al.	1995	–	14 ± 4.0	–	–	–	–	19.6 ± 7.6	–	–	–
Garland et al.	2015	–	–	–	–	–	–	–	–	–	–
Goh et al.	2022	162 ± 9.0	–	23,863 ± 900.0	–	–	198 ± 6.5	–	26,320 ± 700.0	–	–
Guo et al.	2020	–	11 ± 1.0	–	4 ± 0.7	–	–	20 ± 1.8	–	4 ± 1.0	–
Hooper et al.	2009	–	–	–	–	–	–	–	–	–	–
Hou et al.	2021	–	9 ± 0.7	14,503 ± 756.0	–	–	–	15 ± 1.0	16,142 ± 1034.7	–	–
Houdek et al.	2017	176 ± 53.0	4.6 ± 4.1	–	2 ± 1.3	–	211 ± 72.0	5.9 ± 2.4	–	1.9 ± 1.4	–
Inoue et al.	2021	–	1.8 ± 0.8	–	–	–	–	2.8 ± 2.2	–	–	–
Johnston et al.	2011	–	–	–	–	–	–	–	–	–	–
Kamath et al.	2016	134.8 ± 29.1	11.2 ± 3.4	–	–	738.8 ± 519.2	151.5 ± 28.8	15.2 ± 5.8	–	–	943.2 ± 423.0
Kim et al.	2017	172 ± 24.0	10.5 ± 5.8	12,608 ± 2950.0	–	1037 ± 321.0	162 ± 40.0	18.7 ± 8.7	14,910 ± 4080.0	–	1145 ± 518.0
Lindberg‑Larsen et al.	2013	–	6.2 ± 18	–	–	–	–	6.7 ± 10.0	–	–	–
Lorenze et al.	1998	–	10 ± 3.5	26,645 ± 3600	–	535 ± 105.0	–	16 ± 5.0	34,964 ± 5100.0	–	1100 ± 270.0
Martin et al.	2016	130.3 ± 19.9	2.2 ± 0.9	9831 ± 505.1	–	–	139.4 ± 22.0	2.4 ± 0.2	11,544.8 ± 468.4	–	–
Mou et al.	2021	–	–	17,139 ± 1015.0	3 ± 3.9	–	–	–	17,861 ± 1066	0.77 ± 2.0	–
Panchal et al.	2021	–	–	–	–	–	–	–	–	–	–
Partridge et al.	2019	–	8.9 ± 0.7	–	–	–	–	10.4 ± 1.2	–	–	–
Parvizi et al.	2006	131.72 ± 24.4	4.3 ± 2.2	45,900	2.61 ± 1.8	443 ± 152.3	132.3 ± 62.6	8.1 ± 10.3	64,600	3.5 ± 3.5	513 ± 629.0
Poultsides et al.	2017	–	–	–	–	–	–	–	–	–	–
Quadri et al.	2015	273 ± 58.2	8.1 ± 3.2	–	1.2 ± 1.3	–	358 ± 72.6	19.6 ± 5.0	–	2.3 ± 2.6	–
Rasouli et al.	2014	–	–	–	–	–	–	–	–	–	–
Reuben et al.	1998	–	7.6 ± 1.1	24,067 ± 4264.7	–	–	–	14.5 ± 1.8	28,404 ± 1146.3	–	–
Saito et al.	2010	159 ± 32.0	39.6 ± 12	–	–	1018 ± 609.0	179 ± 19.0	60.6 ± 6.5	–	–	1019 ± 358.0
Salvati et al.	1978	–	–	–	–	1944 ± 694	–	–	–	–	2818 ± 900.0
Schlegelmilch et al.	2017	–	–	5735 ± 100.0	–	–	–	–	10,143 ± 346.0	–	–
Seol et al.	2015	–	14.6 ± 8.1	9236 ± 1231.0	3.0 ± 2.6	926.4 ± 341.2	–	25.3 ± 9.8	11,163 ± 1588.4	1.9 ± 2.2	978 ± 389.3
Shih et al.	1985	148 ± 14.0	17.9 ± 6.0	–	–	1202 ± 332.0	245 ± 16.0	27.3 ± 10.9	–	–	1410 ± 230.0
Taheriazam et al.	2019	162 ± 18.0	4.9 ± 1.0	–	1.9 ± 1.3	512 ± 45.0	199.7 ± 16.0	9.8 ± 1.4	–	2.7 ± 2.1	538 ± 390.0
Tan et al.	2019	–	8.7 ± 5.3	19,627 ± 5441.0	–	–	–	12.1 ± 5.6	19,667 ± 5441.0	–	–
Triantafyllopoulos et al.	2016	–	5.2 ± 2.5	–	1.2 ± 1.1	–	–	8.1 ± 2.8	–	1.1 ± 1.2	–
Villa et al.	2019	–	2.6 ± 1.2	–	–	–	–	1.8 ± 1.0	–	–	–

*THA* total hip arthroplasty, *LOS* length of stay, *min* minute, *ml* milliliter

**Table 5:** Postoperative important outcomes reported in each included study

Author	Year	Simultaneous bilateral THA				Staged bilateral THA			
		Revision (n)	Readmission (n)	Postoperative LLD (mm)	Postoperative HHS	Revision (n)	Readmission (n)	Postoperative LLD (mm)	Postoperative HHS
Agarwal et al.	2016	–	–	10 ± 3.0	92.3 ± 1.2	–	–	9 ± 3.5	90.8 ± 1.1
Aghayev et al.	2010	–	–	–	94.2 ± 2.0	–	–	–	91 ± 3.0
Alfaro-Adrián et al.	1999	5	–	–	–	7	–	–	–
Berend et al.	2007	–	13	–	–	–	1	–	–
Bhan et al.	2006	3	–	4.5 ± 4.4	82 ± 5.0	3	–	5.3 ± 4.6	83.5 ± 6.0
Brown et al.	2017	–	–	3.5 ± 2.7	–	–	–	3.8 ± 3.0	–
Calabro et al.	2020	121	–	–	–	500	–	–	–
Eggli et al.	1995	–	–	2.2 ± 1.8	–	–	–	2.2 ± 1.4	–
Garland et al.	2015	240	–	–	–	4897	–	–	–
Goh et al.	2022	–	–	–	–	–	–	–	–
Guo et al.	2020	–	14	–	–	–	5	–	–
Hooper et al.	2009	–	–	–	–	–	–	–	–
Hou et al.	2021	–	–	–	–	–	–	–	–
Houdek et al.	2017	7	9	–	–	13	15	–	–
Inoue et al.	2021	–	–	–	–	–	–	–	–
Johnston et al.	2011	1	16	–	78.9 ± 10.3	8	142	–	82.2 ± 13.4
Kamath et al.	2016	–	–	1.1 ± 1.8	–	–	–	1.3 ± 1.7	–
Kim et al.	2017	2	–	2.1 ± 2.0	95.9 ± 4.8	4	–	4.3 ± 3.2	90.7 ± 8.2
Lindberg‑Larsen et al.	2013	–	2	–	–	–	33	–	–
Lorenze et al.	1998	–	–	–	–	–	–	–	–
Martin et al.	2016	–	–	–	–	–	–	–	–
Mou et al.	2021	–	–	4.8 ± 3.9	84 ± 2.8	–	–	4.5 ± 3.1	83.4 ± 2.0
Panchal et al.	2021	–	–	–	–	–	–	–	–
Partridge et al.	2019	–	55	–	–	–	198	–	–
Parvizi et al.	2006	0	–	–	91 ± 3.0	3	–	–	89 ± 3.0
Poultsides et al.	2017	–	–	–	–	–	–	–	–
Quadri et al.	2015	0	–	–	–	1	–	–	–
Rasouli et al.	2014	–	–	–	–	–	–	–	–
Reuben et al.	1998	–	–	–	–	–	–	–	–
Saito et al.	2010	–	–	–	87.8 ± 4.0	–	–	–	87.3 ± 2.6
Salvati et al.	1978	–	–	–	–	–	–	–	–
Schlegelmilch et al.	2017	–	–	–	–	–	–	–	–
Seol et al.	2015	–	–	–	96.4	–	–	–	94.8
Shih et al.	1985	–	–	–	–	–	–	–	–
Taheriazam et al.	2019	0	–	–	84.1 ± 3.0	0	–	–	82.6 ± 3.1
Tan et al.	2019	1	–	–	–	1	–	–	–
Triantafyllopoulos et al.	2016	–	–	–	–	–	–	–	–
Villa et al.	2019	–	–	–	–	–	–	–	–

*THA* total hip arthroplasty, *HHS* Harris hip score, *LLD* leg length discrepancy, *n* number

### Systematic review of heterogeneous data

Based on 12 studies [18–20, 24, 25, 29, 34, 37, 38, 41, 42, 54], the mean operation time was 171.4 min for simBTHA and 191.4 min for stgBTHA. Cumulative operation time for both surgeries in stgBTHA was longer than simBTHA operation time in all studies except the study by Kim et al. [42]. Although postoperative Western Ontario and McMaster Universities Arthritis Index (WOMAC) scores were reported to be similar between the two groups [35], two studies reported significantly higher scores of Oxford Hip Scores [56] or EuroQoL-5D index [42] in simBTHA compared to stgBTHA. In contrast, another study by Kamath et al. [37] stated no statistical difference between the two groups in mentioned functional outcomes. Functional recovery was faster in simBTHA, as walking without support started earlier [36] and walking capacity was better postoperatively [21, 28]. Rates of home-discharged patients for stgBTHA were higher in all studies [25, 26, 40, 41, 43, 49, 54].

For 90-day mortality, systemic complications, operation cost, LOS, blood loss, blood transfusion rate, HHS, LLD, and high heterogeneity existed between studies (*I*^2^ ranged from 59.909 to 99.729%). Begg’s funnel plots are shown in Additional file 3.

## Discussion

SimBTHA has continued to attract attention since Charnley first introduced this type of orthopedic surgery. Many studies comparing simBTHA and stgBTHA have been conducted since then but, due to small sample size or other undetermined possible reasons, failed to obtain a definite conclusion. We conducted a comprehensive systematic review and meta-analysis of 38 comparative studies enrolling 104,151 patients. Findings of this updated meta-analysis generally concur and further extend that of previous reviews on the topic, providing several relevant results that have not been previously addressed.

### Mortality and complications

The combined 90-day mortality rate was 0.22% for simBTHA and 1.57% for stgBTHA. Nonetheless, the 90-day mortality analysis failed to show any significant difference between the two groups. Since most included articles were retrospective studies, we should interpret the present results with caution. Previous studies have also posed no significant difference in mortality rate between the two groups [7, 32, 33, 48, 57].

Periprosthetic joint infection (PJI), as an uncommon complication of THA [58], can incur costs for the patient and healthcare system [59]. PJI can also lead to secondary surgery and even death [60]. No significant difference was observed regarding the PJI rate between the two groups. However, our results contrast with the previous review [7], which indicated a significantly higher infection rate in one-stage versus two-stage. Shao et al. [7] computed the risk in the cumulative number of superficial and deep infection cases, so their effect on subsequent procedures on hospitalization might be diverse. The overall PJI rate was 0.91% in the simBTHA group and 0.87% in the stgBTHA group. The overall PJI rate for both groups was higher than in previous studies [39, 61].

We investigated periprosthetic fracture between the two groups, and contrary to previous studies [5, 7, 41, 51], the incidence of fracture in simBTHA was higher than in stgBTHA. The unanticipated increased fracture risk in simBTHA can be attributed to the cemented or cementless fixation [62] and operation time in a single surgery. As in the previous meta-analyses [5, 7, 63], no clinically significant difference was seen in the occurrence of dislocation between the two groups in our study.

We found a significantly lower risk of DVT in simBTHA compared to stgBTHA. This finding is consistent with previous studies [7, 8]. Lower activity levels in stgBTHA due to pain in the contralateral hip can justify the elevated risk of DVT in stgBTHA [64]. Despite simBTHA patients having an associated lower risk of DVT, we observed an increased risk of PE in simBTHA compared to stgBTHA. Still, other investigations revealed no difference [5, 7, 57] or an elevated risk of PE in StgBTHA [8] PE, consuming a huge part of medical resources [65], can yield in-hospital and post-discharge mortality [66]. A large-scale data registry study by partridge et al. [48] suggested that simBTHA is associated with a greater risk of developing PE. This study included more than half of our study population and maybe has shifted the results toward itself. However, the quality of this study was high and might not have imposed bias on the results. We should consider that pharmacological thromboprophylaxis can reduce thromboembolic events [67], and many risk factors affect PE incidence [68].

The stgBTHA was associated with a higher risk for postoperative pulmonary complications. Malcolm et al. also reported a 1.42% respiratory complication rate for THA, similar to the simBTHA group in our study [69]. In our study, the pulmonary complications rate in simBTHA and stgBTHA was 1.69% and 2.38%, respectively.

On the other hand, a higher risk of systemic and local complications in the stgBTHA was evidenced. Similar results were reported by Aghayev et al. [28]. Poultsides et al. [43] and Guo et al. [47] also presented that the rate of systemic complications in simBTHA was lower than in stgBTHA.

### Other outcomes

Combining the results of 10 studies revealed no significant differences in revision rate between the simBTHA and stgBTHA. Our findings are compatible with the previous study [46] published on this topic. Another study by Garland et al. [33] indicated a slightly higher risk of revision for stgBTHA. There were no significant differences among simBTHA and stgBTHA concerning readmission rates in keeping with previous studies [41, 47, 48].

Our research shows that simBTHA is superior to stgBTHA in terms of cumulative operation time, hospital cost, and LOS. The simBTHA surgery is performed in one session, while the stgBTHA surgery is performed in two sessions. Undergoing two operations, which obviously has a longer cumulative operation time, means a more extended anesthesia period which is correlated with increased risk of infection [70], venous thromboembolism (VTE) [71], neurologic deficit [72], revision, intraoperative blood loss, transfusion, and other critical adverse events [73, 74]. Operation time is a potentially modifiable risk factor that engages surgeons and healthcare systems interested in quality improvement. Sodhi et al. [75] saw that operation time is significantly associated with LOS, and LOS has also been a major driver of cost in THA [76]. Mean LOS for simBTHA was 4.8 days less than stgBTHA, which can justify more costs and complications in stgBTHA. However, operation time is varied by various factors such as operating technique, surgery approach, general or epidural anesthesia, patient's demographics, and surgeon's experience. Although almost all studies demonstrated a lower cost, and LOS in simBTHA, researchers utilized various methods to calculate these data. Therefore, high heterogeneity was observed in the pooled data.

The aggregate results of our study indicated that simBTHA outperformed stgBTHA in reducing perioperative total blood loss. Previous studies also showed a higher cumulative blood loss in stgBTHA compared to simBTHA [5, 18, 24]. Interestingly, in this meta-analysis, despite a lower total blood loss in simBTHA, analysis of transfusion units did not show any significant difference between the two groups. It should be taken into account that indications for blood transfusion in different studies were not the same. Another reason for similar rates of blood transfusion could be the interval between two operations in stgBTHA that provides enough time for hematopoiesis. In a retrospective study [39], comparing infection rates after THA, blood transfusion has found to be a powerful risk factor for PJI, and patients who underwent simBTHA had a higher blood transfusion rate than stgBTHA. In contrast, another study by Parvizi et al. [25] revealed that the cumulative blood transfusion was lower in simBTHA compared with stgBTHA. As higher blood loss is accompanied by more need for blood transfusion in which itself is associated with a higher risk for infection [77], immunosuppression [78], and even death [79], blood loss stands as a significant concern in major orthopedic surgeries [80].

Although the pooled results of analysis favored simBTHA in terms of the postoperative HHS, but a 1.37 point improvement is not clinically significant based on the prior evidence [81]. Kim et al. [42] found that the mean postoperative HHS was significantly higher in simBTHA than in stgBTHA, and they mentioned that better functional outcomes in simBTHA could be because of the accuracy of surgery, earlier starting rehabilitation for both operated hips, and reduced time lost from work in a simultaneous procedure. The diversity of functional outcome measure types did not allow us concluding precisely regarding hip joint function. Using a comprehensive and unified tool that includes important items for hip joint function evaluation can help us decide more precisely which type of surgery is appropriate for specific situation.

Concomitant to our results, several studies have exhibited no difference in LLD between simBTHA and stgBTHA [36, 37, 40]. However, LLD can yield patient dissatisfaction after THA [82]. It also has been indicated that LLD can worsen functional outcomes such as Oxford Hip Score [83].

The strength points of this meta-analysis comprise peer-reviewed comparative studies and a rigorous assessment of the methodological quality of the currently available data. This study enhanced the power to compare the clinical outcomes of simBTHA and stgBTHA through more excellent details. With respect to the previous meta-analysis [8], we used explicit exclusion and inclusion criteria. We also utilized a robust search strategy spanned multiple databases, yielding 38 published studies on the topic, twice the number of included studies in the previous meta-analysis.

Our study has several potential limitations. First, due to the limited number of RCTs, we included non-RCTs, too. As we know, retrospective studies vary in terms of quality, making our study susceptible to bias and confounding. Second, we also excluded non-English studies, which may cause language bias in our research. Third, lacking a specific definition for some outcomes like operation time and variety of measurements may bias our findings. Fourth, most of the studies did not report outcomes according to surgical approach, method of anesthesia, use of antibiotics and thrombosis prophylaxis, primary diagnosis, and demographic data. Although our goal was not to compare these data, they could have influenced the accuracy of our results. Fifth, some studies did not contain raw data for pooled analyses. Although we tried to contact the authors, we could not get these data. Sixth, each study’s criteria for blood transfusion were different or not mentioned. Seventh, the number of participants varied considerably among the included studies, ranging from 15 to 42,238. Eighth, National registry data studies have some missing information about patients and these studies may also underestimate complications rates which could have influenced the final result. Ninth, follow-up periods were heterogeneous among studies. Tenth, HHS measurements were done at different times, which might have biased our results. At last, we combined different complications to obtain two categories: systemic and local. However, some studies avoided reporting complications separately, so they put together all of them without paying attention to the different severity, which limits the conclusion's reliability.

## Conclusion

Taken together, this meta-analysis demonstrated that simultaneous and staged THA have similar 90-day mortality, dislocation, and PJI rates. A statically significant risk reduction was identified in DVT, pulmonary, systemic, and local complications in the simBTHA group. Interestingly, stgBTHA is more promising in terms of PE and fracture rate. The present study also revealed that simBTHA is associated with lower total blood loss, length of stay, and total surgery cost. Reduced length of hospital stay and total surgery cost as essential advantages of simBTHA compared to stgBTHA may attract healthcare providers' and policy-makers' attention. After all, simBTHA remains noninferior to the stgBTHA in most postoperative outcomes. Anyhow, we recommend that well-designed randomized controlled trials should be conducted to elucidate the advantages of each surgery in order to help surgeons choose the proper surgical method hinged on their point of view and patient's benefits.

## Supplementary Information

**Additional file 1.** PRISMA Checklist.

**Additional file 2.** Search String.

**Additional file 3.** Begg’s funnel plots and Egger’s regression test.

## Data Availability

The datasets used and/or analyzed during the current study are available from the corresponding author on reasonable request.

## Abbreviations

THA: Total hip arthroplasty
simBTHA: Simultaneous bilateral THA
stgBTHA: Staged bilateral THA
DVT: Deep vein thrombosis
PE: Pulmonary embolism
Venous thromboembolism: Venous thromboembolism
BMI: Body mass index
ASA Classification: American Society of Anesthesiology
LOS: Length of hospital stay
HHS: Harris hip score
WOMAC: The Western Ontario and McMaster Universities Arthritis Index
LLD: Limb length discrepancy
VTE: Venous thromboembolism
PJI: Periprosthetic joint infection
