# Supplementary material for: Simultaneous versus staged bilateral total hip arthroplasty: a systematic review and meta-analysis
Source: J Orthop Surg Res. 2022 Aug 13;17:392. doi: 10.1186/s13018-022-03281-4 (PMC9375332; doi:10.1186/s13018-022-03281-4)
Supplement: Supplementary file 2 — Additional file 2. Search String. [file 13018_2022_3281_MOESM2_ESM.docx]

**Simultaneous versus staged bilateral total hip arthroplasty; a systematic review and meta-analysis**

***Journal of Orthopaedic Surgery and Research***

***Search string***

***MEDLINE:***

((total hip arthroplasty[MeSH Terms]) OR (Total hip arthroplasty[Title/Abstract]) OR (“Total Hip Replacement”[Title/Abstract]) OR (Hip Replacement Arthroplasty[Title/Abstract]) OR (Hip Prosthesis Implantation[Title/Abstract]) OR (Microinvasive Total Hip Arthroplasty[Title/Abstract]) OR (hip arthroplasty[Title/Abstract])) AND ((Bilateral[Title/Abstract]) OR (one-stage*[Title/Abstract]) OR (two-stage*[Title/Abstract]) OR (1-stage*[Title/Abstract]) OR (2-stage*[Title/Abstract]) OR (single-stage[Title/Abstract]) OR (Simultaneous[Title/Abstract]) OR (stage*[Title/Abstract]) OR (sequential[Title/Abstract]) OR (single-anesthetic[Title/Abstract]) OR (single anesthesia[Title/Abstract]) OR (single hospital admission[Title/Abstract]))

***SCOPUS:***

TITLE-ABS-KEY(“Total hip arthroplasty” OR “Total Hip Replacement” OR “Hip Replacement Arthroplasty” OR “Hip Prosthesis Implantation” OR “Microinvasive Total Hip Arthroplasty” OR “hip arthroplasty”) AND TITLE-ABS-KEY(“Bilateral” OR “one-stage*” OR “single-stage*” OR “two-stage*” OR “1-stage*” OR “2-stage*” OR “Simultaneous” OR “stage*” OR “sequential” OR “single-anesthetic“ OR “single anesthesia” OR “single hospital admission”)

***WOS:***

All=(“Total hip arthroplasty” OR “Total Hip Replacement” OR “Hip Replacement Arthroplasty” OR “Hip Prosthesis Implantation” OR “Microinvasive Total Hip Arthroplasty” OR “hip arthroplasty”) AND All=(“Bilateral” OR “one-stage*” OR “single-stage*” OR “two-stage*” OR “1-stage*” OR “2-stage*” OR “Simultaneous” OR “stage*” OR “sequential” OR “single-anesthetic“ OR “single anesthesia” OR “single hospital admission”)

***EMBASE:***

#1 ‘hip replacement’/exp OR ‘hip arthroplasty’/exp

#2 ‘Total hip arthroplasty’:ti,ab,kw OR ‘Total Hip Replacement’:ti,ab,kw OR ‘Hip Replacement Arthroplasty’:ti,ab,kw OR ‘Hip Prosthesis Implantation’:ti,ab,kw OR ‘Microinvasive Total Hip Arthroplasty’:ti,ab,kw OR ‘hip arthroplasty’:ti,ab,kw

#3 #1 OR #2

#4 ‘Bilateral’:ti,ab,kw OR ‘one-stage*’:ti,ab,kw OR ‘single-stage*’:ti,ab,kw OR ‘two-stage*’:ti,ab,kw OR ‘1-stage*’:ti,ab,kw OR ‘2-stage*’:ti,ab,kw OR ‘Simultaneous’:ti,ab,kw OR ‘stage*’:ti,ab,kw OR ‘sequential’:ti,ab,kw OR ‘single-anesthetic’:ti,ab,kw OR ‘single anesthesia’:ti,ab,kw OR ‘single hospital admission’:ti,ab,kw

#5 #3 AND #4
