# Supplementary material for: Simultaneous versus staged bilateral total hip arthroplasty: a systematic review and meta-analysis
Source: J Orthop Surg Res. 2022 Aug 13;17:392. doi: 10.1186/s13018-022-03281-4 (PMC9375332; doi:10.1186/s13018-022-03281-4)
Supplement: Supplementary file 3 — Additional file 3. Begg’s funnel plots and Egger’s regression test. [file 13018_2022_3281_MOESM3_ESM.pdf]

Simultaneous versus staged bilateral total hip arthroplasty; a systematic review and meta-analysis

Journal of Orthopaedic Surgery and Research

Supplementary Materials

Funnel plot and Egger's regression test for DVT

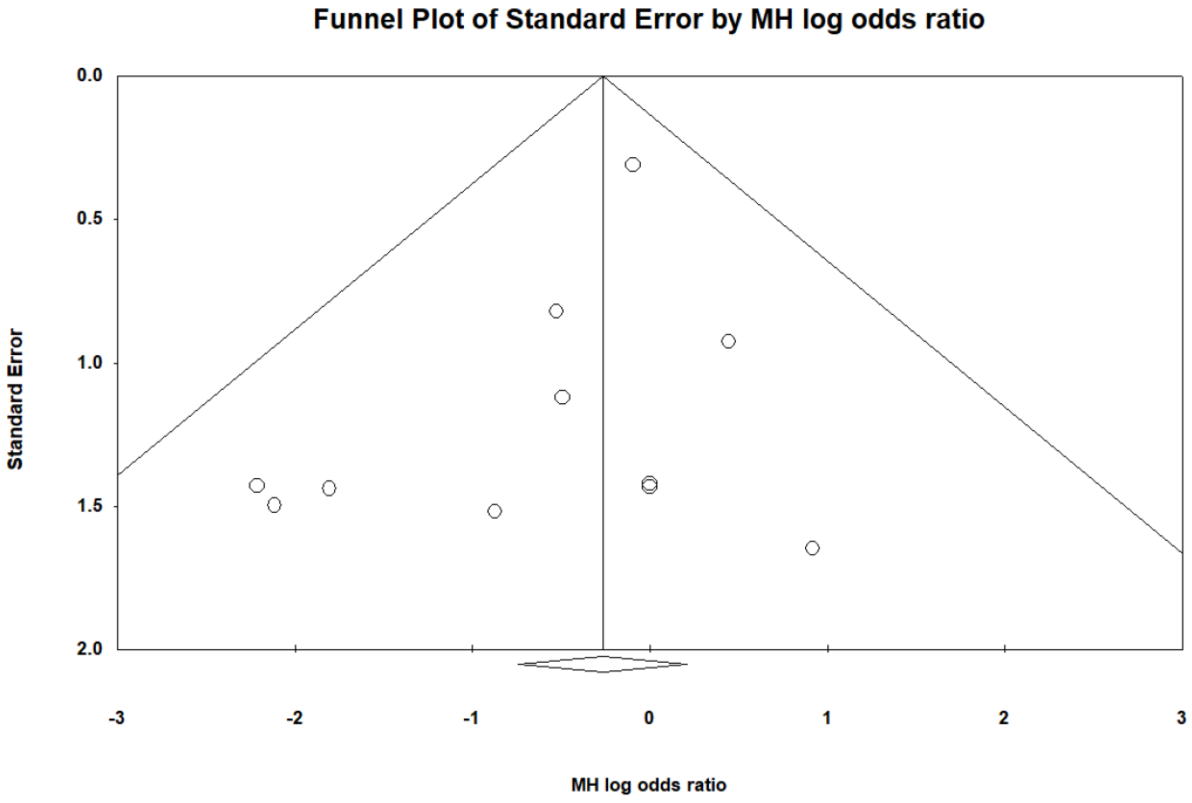

Egger's regression intercept

Intercept	-0.57818
Standard error	0.38837
95% lower limit (2-tailed)	-1.45673
95% upper limit (2-tailed)	0.30038
t-value	1.48872
df	9.00000
P-value (1-tailed)	0.08537
P-value (2-tailed)	0.17074

Funnel plot and Egger's regression test for PE

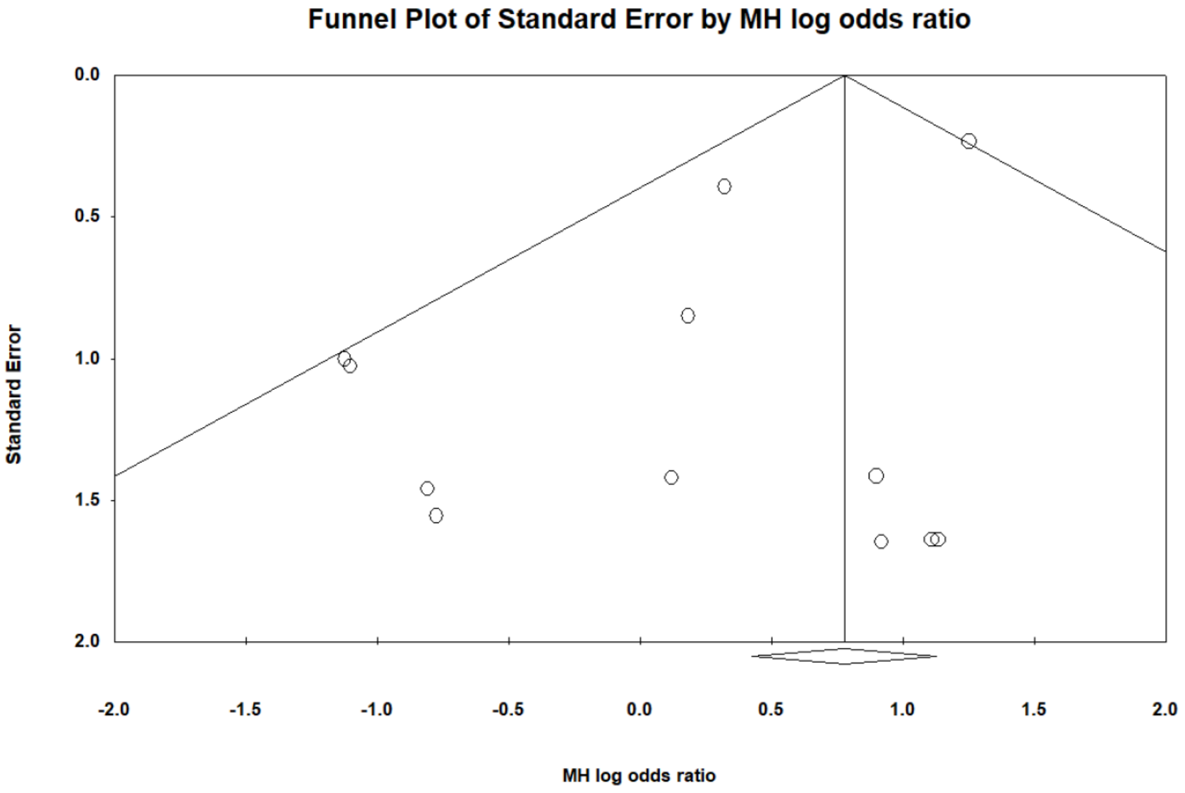

Egger's regression intercept

Intercept	-1.05056
Standard error	0.42985
95% lower limit (2-tailed)	-2.00832
95% upper limit (2-tailed)	-0.09280
t-value	2.44404
df	10.00000
P-value (1-tailed)	0.01730
P-value (2-tailed)	0.03461

Funnel plot and Egger's regression test for Pulmonary complications

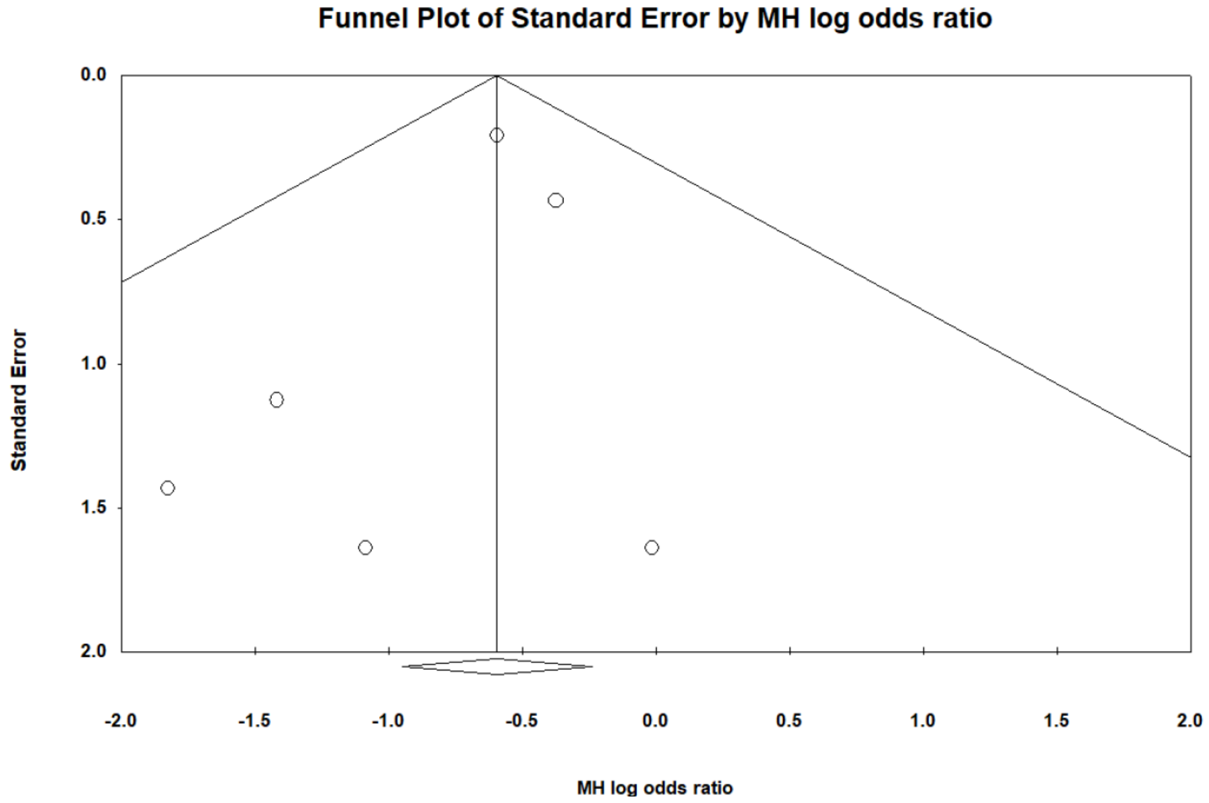

Egger's regression intercept

Intercept	-0.36626
Standard error	0.35168
95% lower limit (2-tailed)	-1.34267
95% upper limit (2-tailed)	0.61016
t-value	1.04146
df	4.00000
P-value (1-tailed)	0.17823
P-value (2-tailed)	0.35647

Funnel plot and Egger's regression test for Systemic complications

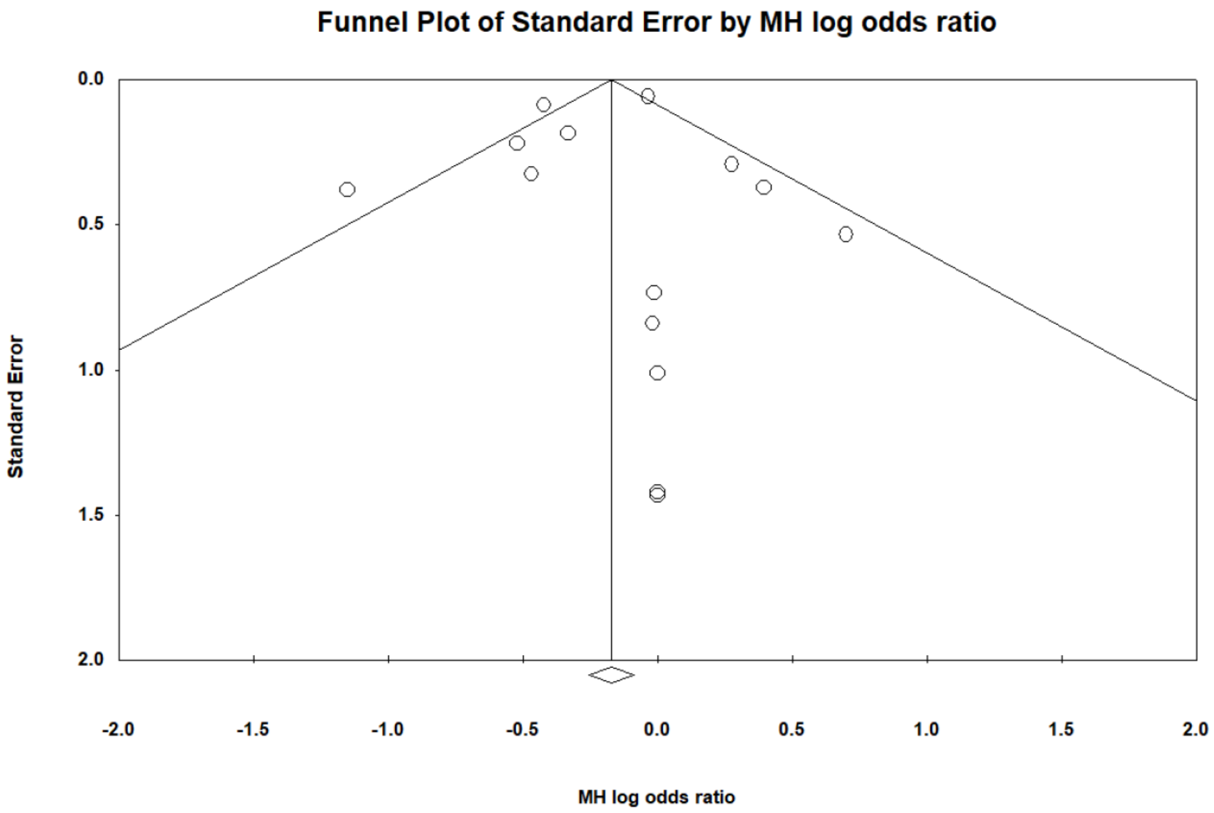

Egger's regression intercept

Intercept	-0.13225
Standard error	0.58769
95% lower limit (2-tailed)	-1.41272
95% upper limit (2-tailed)	1.14823
t-value	0.22502
df	12.00000
P-value (1-tailed)	0.41287
P-value (2-tailed)	0.82575

Funnel plot and Egger's regression test for Local complications

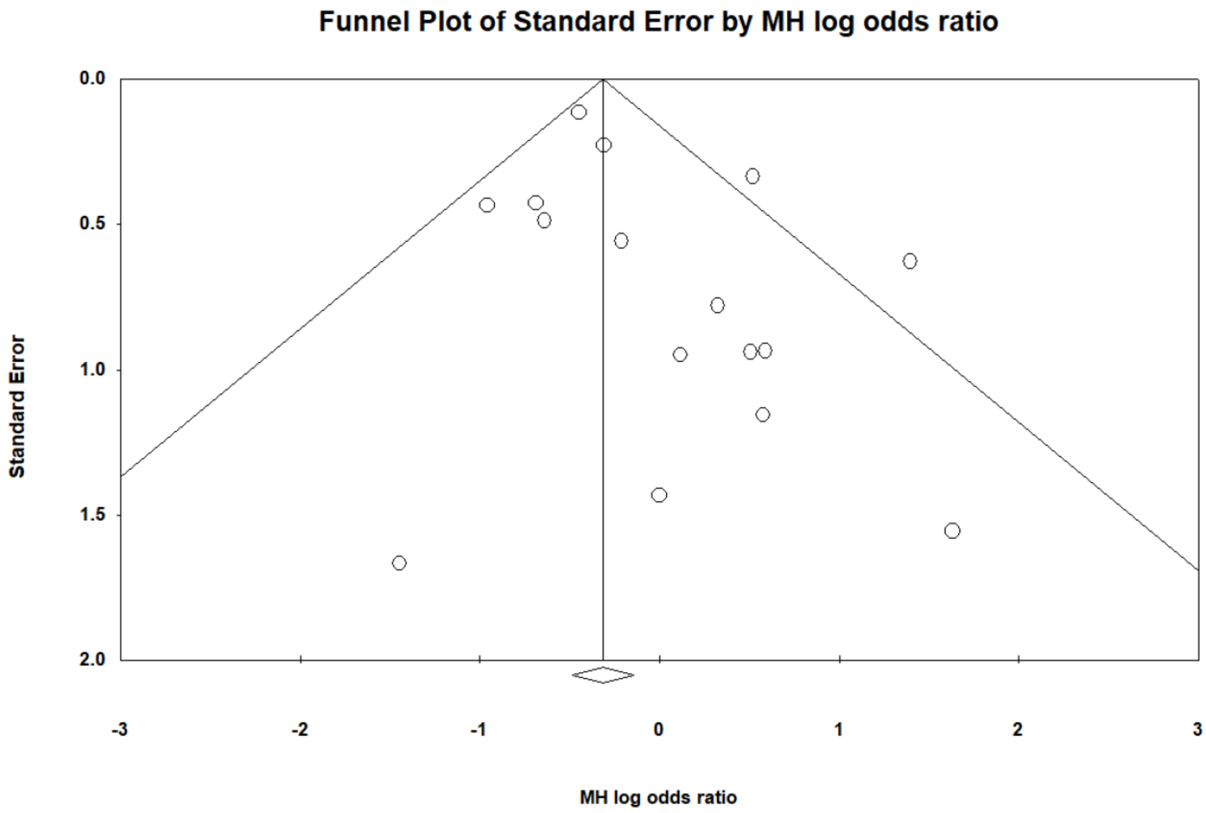

Egger's regression intercept

Intercept	0.78805
Standard error	0.42490
95% lower limit (2-tailed)	-0.12327
95% upper limit (2-tailed)	1.69936
t-value	1.85467
df	14.00000
P-value (1-tailed)	0.04241
P-value (2-tailed)	0.08482

Funnel plot and Egger's regression test for PJI

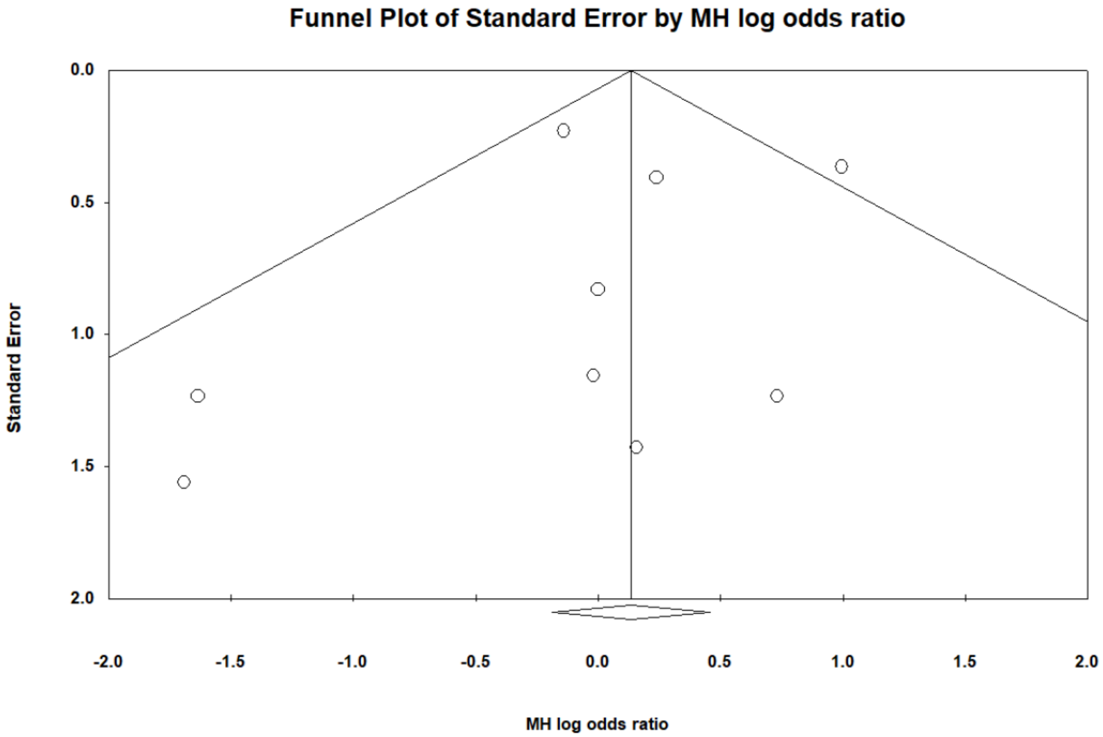

Egger's regression intercept

Intercept	-0.30833
Standard error	0.67740
95% lower limit (2-tailed)	-1.91014
95% upper limit (2-tailed)	1.29347
t-value	0.45517
df	7.00000
P-value (1-tailed)	0.33138
P-value (2-tailed)	0.66277

Funnel plot and Egger's regression test for Fracture

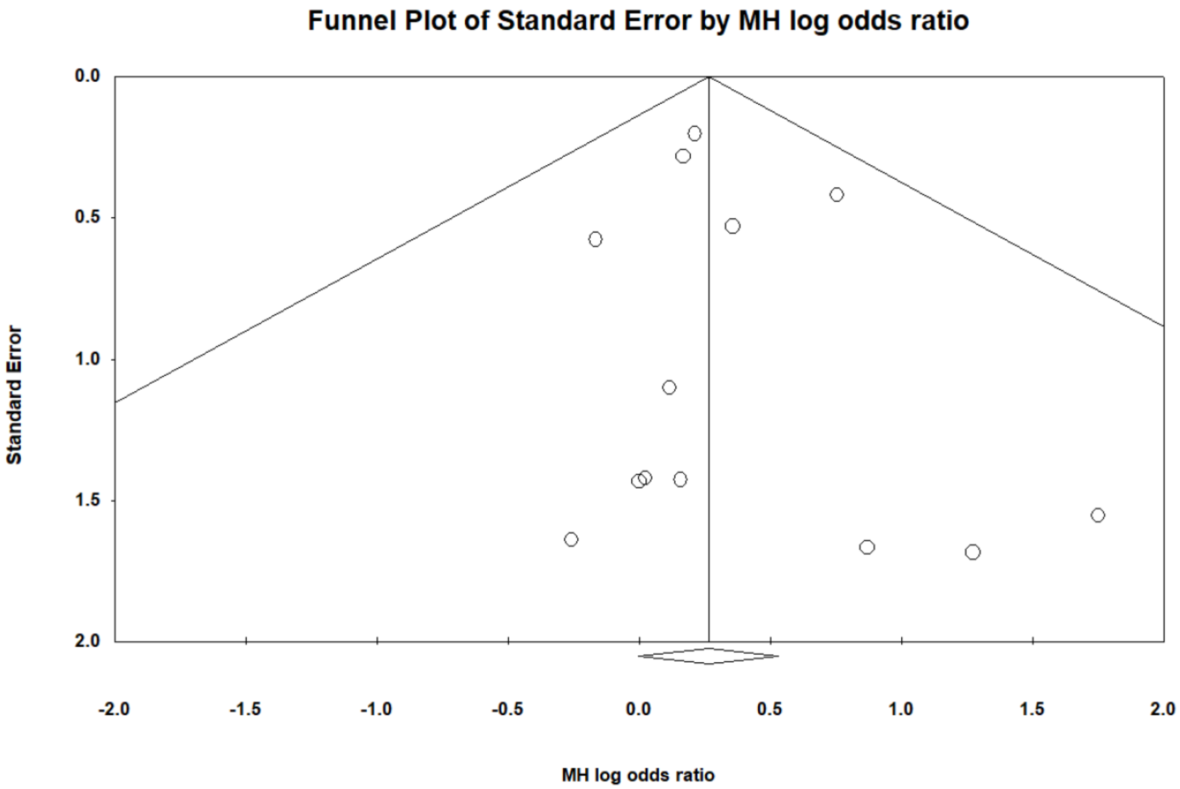

Egger's regression intercept

Intercept	0.19336
Standard error	0.24096
95% lower limit (2-tailed)	-0.33699
95% upper limit (2-tailed)	0.72371
t-value	0.80246
df	11.00000
P-value (1-tailed)	0.21964
P-value (2-tailed)	0.43928

Funnel plot and Egger's regression test for Dislocation

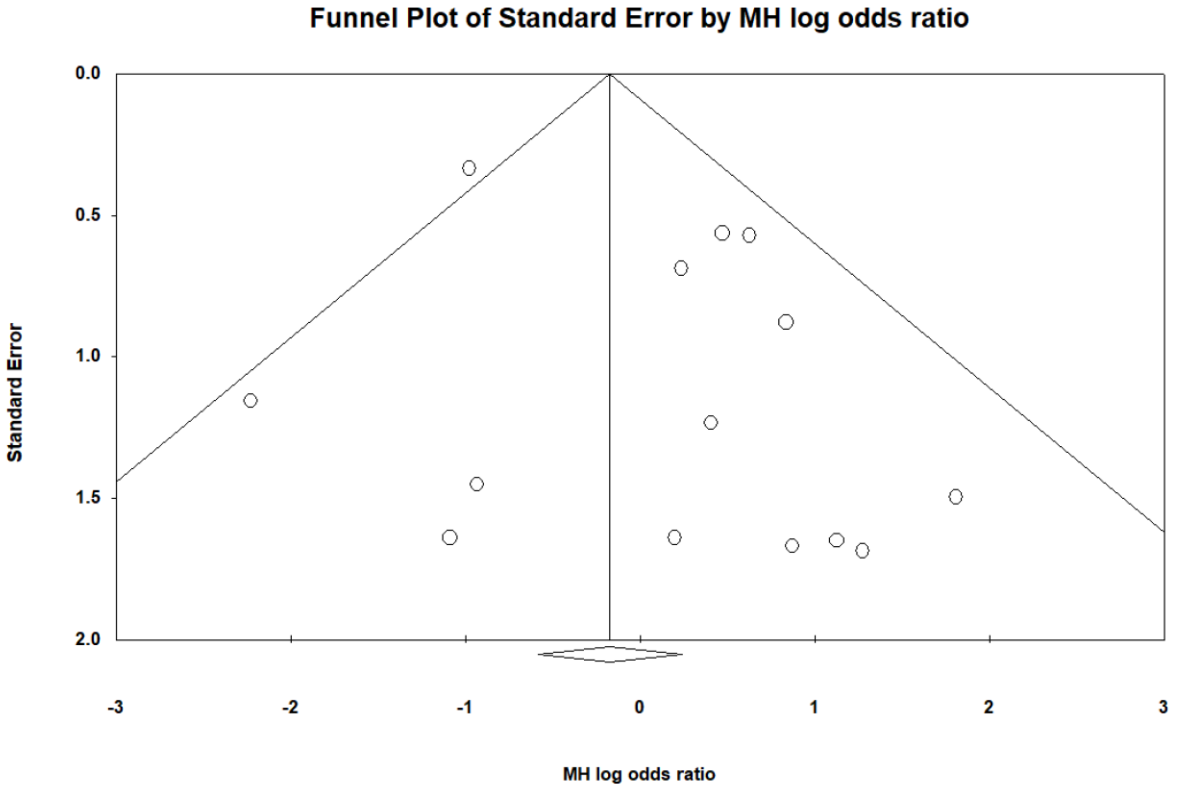

Egger's regression intercept

Intercept	0.85617
Standard error	0.57406
95% lower limit (2-tailed)	-0.39459
95% upper limit (2-tailed)	2.10693
t-value	1.49144
df	12.00000
P-value (1-tailed)	0.08083
P-value (2-tailed)	0.16166

Funnel plot and Egger's regression test for Mortality

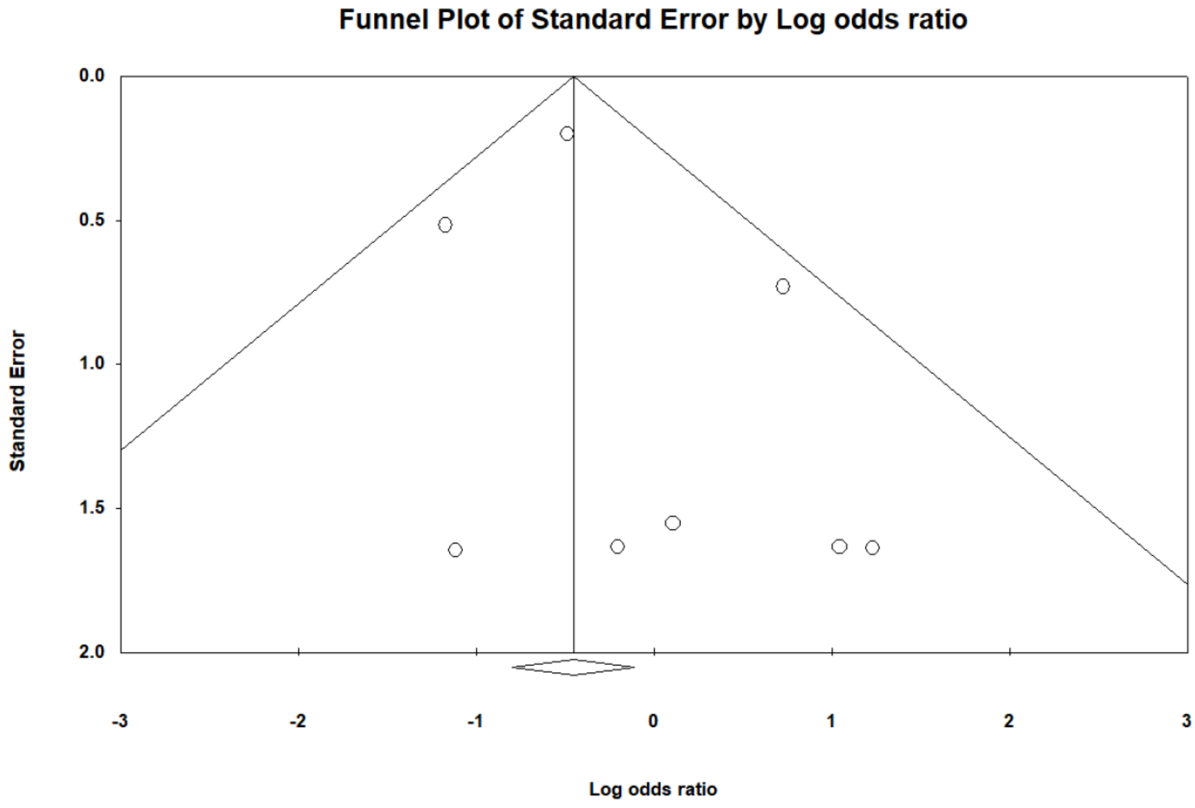

Egger's regression intercept

Intercept	0.53165
Standard error	0.48443
95% lower limit (2-tailed)	-0.65370
95% upper limit (2-tailed)	1.71699
t-value	1.09747
df	6.00000
P-value (1-tailed)	0.15726
P-value (2-tailed)	0.31451

Funnel plot and Egger's regression test for LOS

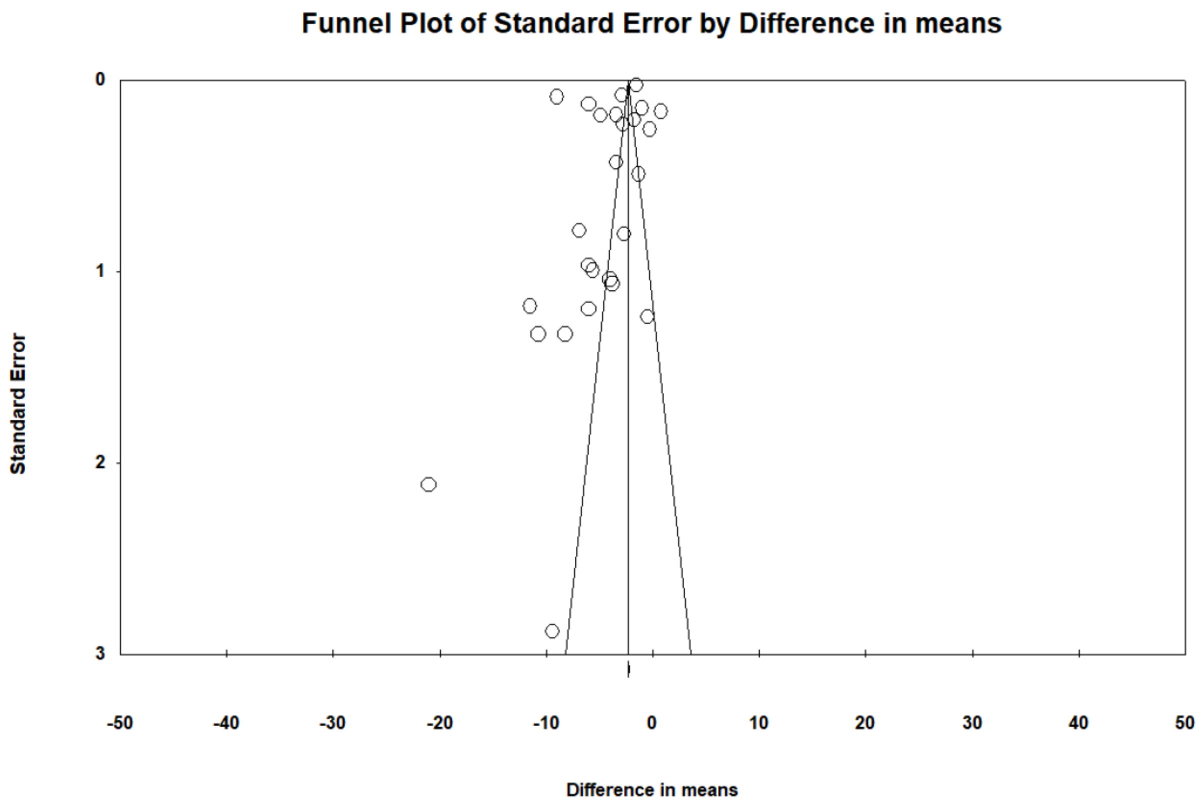

Egger's regression intercept

Intercept	-6.42221
Standard error	4.32634
95% lower limit (2-tailed)	-15.35133
95% upper limit (2-tailed)	2.50691
t-value	1.48444
df	24.00000
P-value (1-tailed)	0.07535
P-value (2-tailed)	0.15071

Funnel plot and Egger's regression test for Operation Time

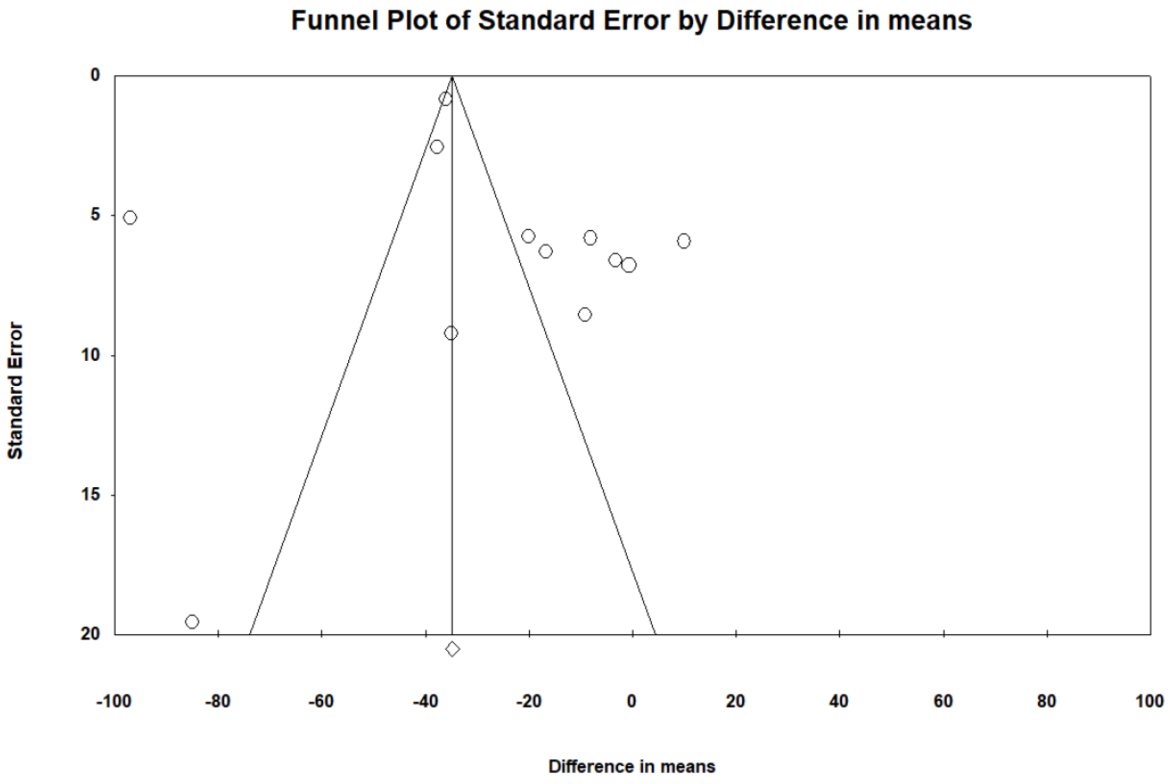

Egger's regression intercept

Intercept	1.85934
Standard error	2.02077
95% lower limit (2-tailed)	-2.64321
95% upper limit (2-tailed)	6.36189
t-value	0.92012
df	10.00000
P-value (1-tailed)	0.18959
P-value (2-tailed)	0.37917

Funnel plot and Egger's regression test for Total Blood Loss

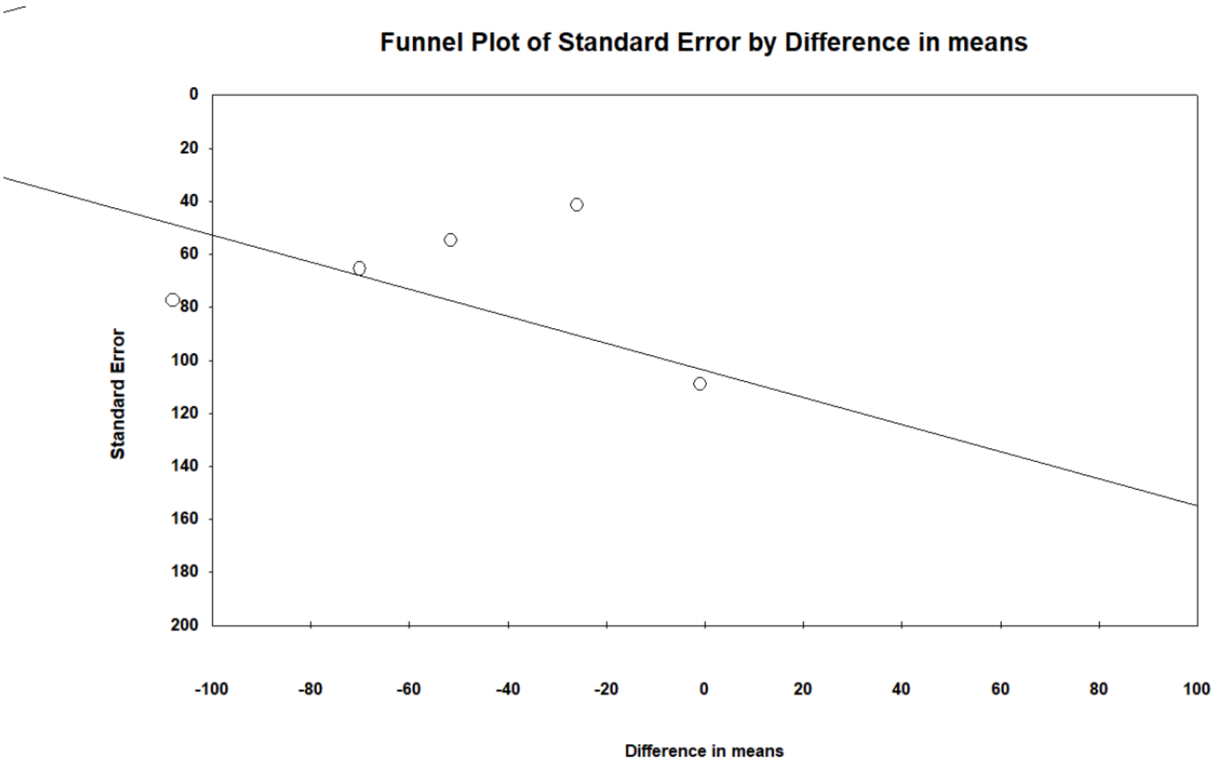

Egger's regression intercept

Intercept	-1.88343
Standard error	2.24470
95% lower limit (2-tailed)	-6.88494
95% upper limit (2-tailed)	3.11808
t-value	0.83905
df	10.00000
P-value (1-tailed)	0.21052
P-value (2-tailed)	0.42104

Funnel plot and Egger's regression test for Blood Transfusion need

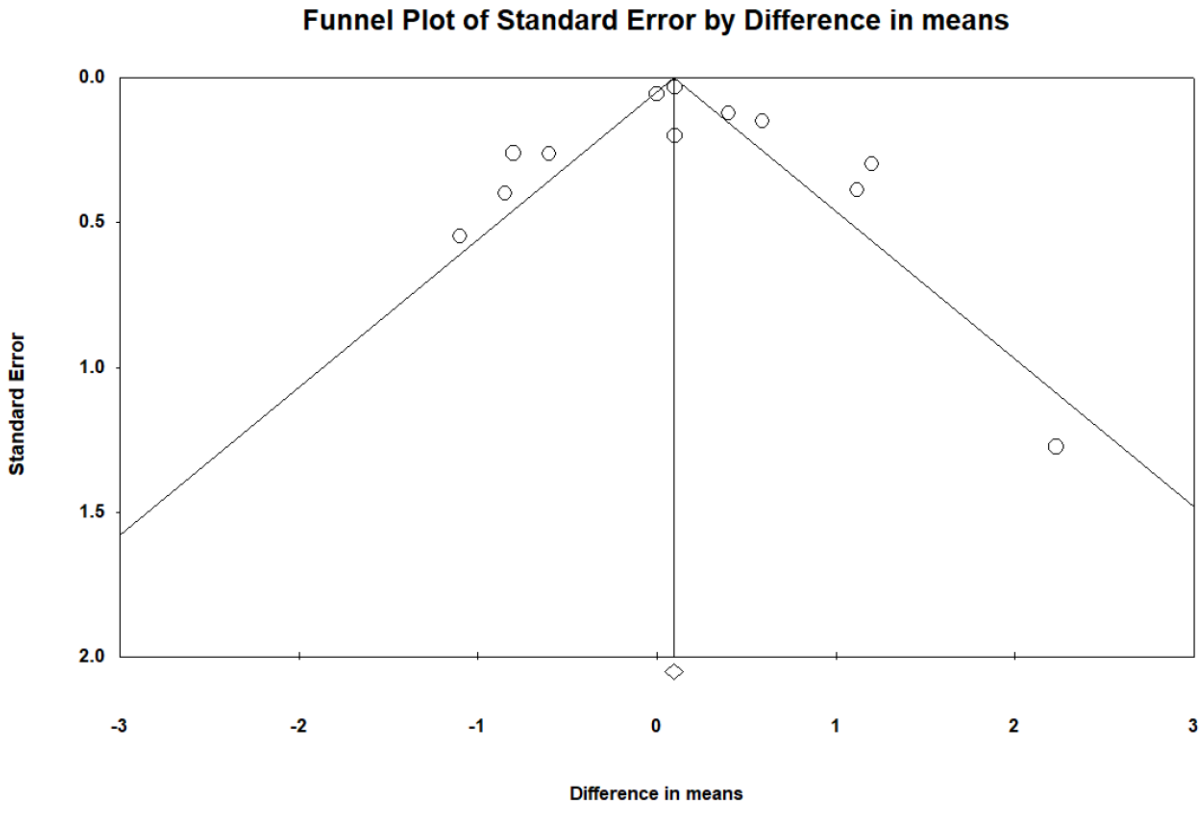

Egger's regression intercept

Intercept	0.22640
Standard error	1.03344
95% lower limit (2-tailed)	-2.07624
95% upper limit (2-tailed)	2.52905
t-value	0.21908
df	10.00000
P-value (1-tailed)	0.41550
P-value (2-tailed)	0.83100

Funnel plot and Egger's regression test for Revision

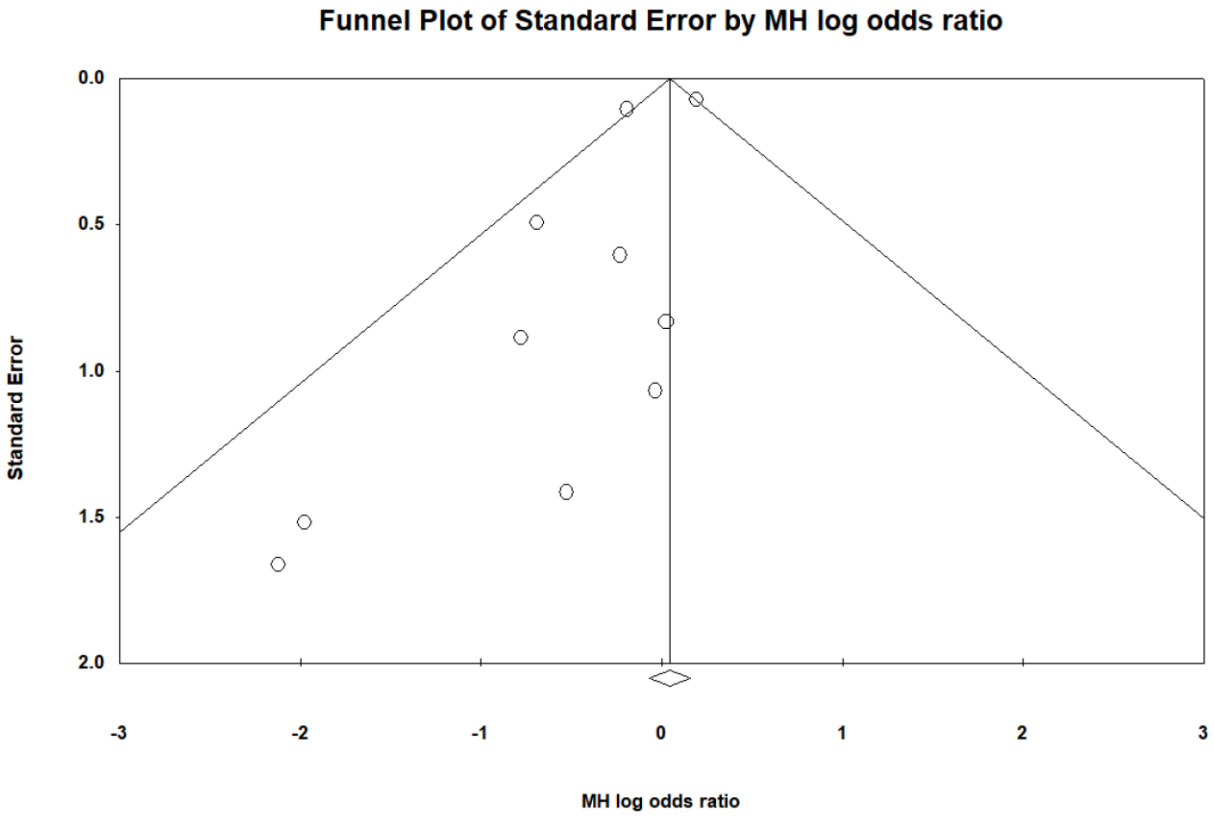

Egger's regression intercept

Intercept	-0.95695
Standard error	0.44716
95% lower limit (2-tailed)	-1.98811
95% upper limit (2-tailed)	0.07422
t-value	2.14003
df	8.00000
P-value (1-tailed)	0.03239
P-value (2-tailed)	0.06477

Funnel plot and Egger's regression test for Readmission

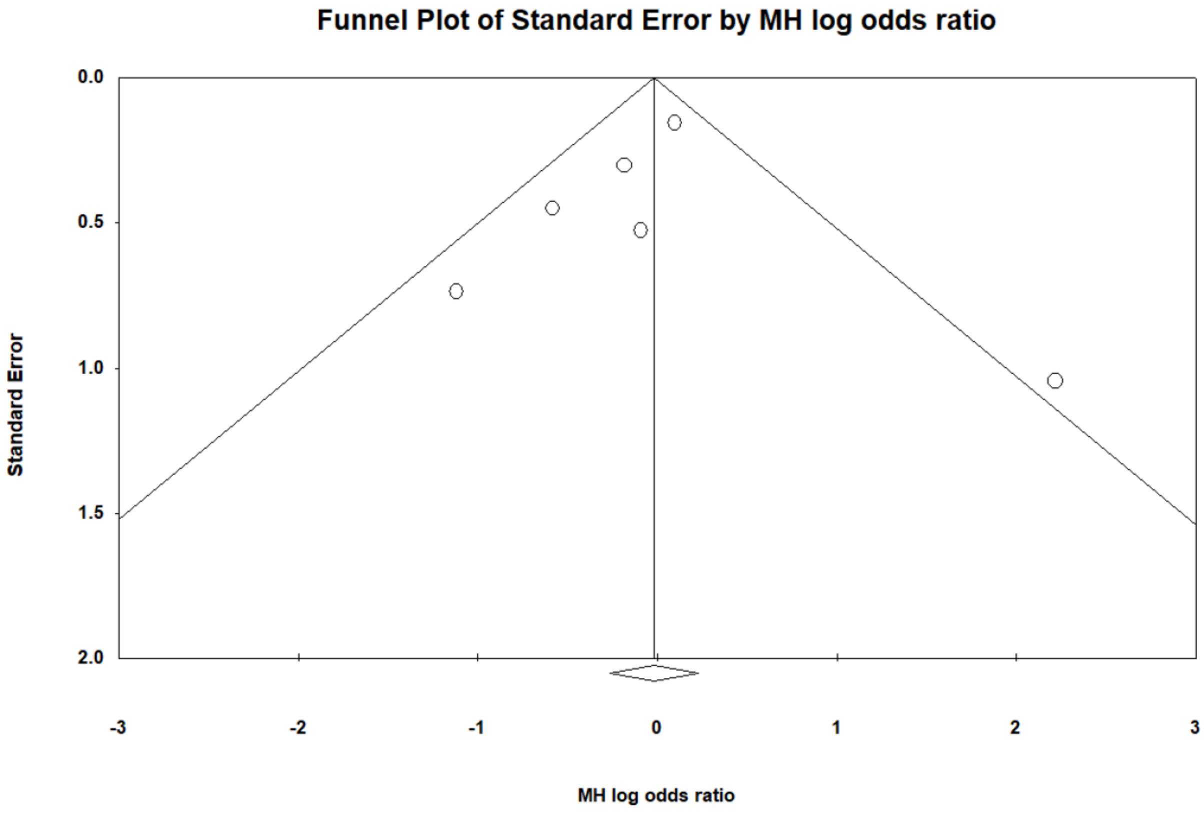

Egger's regression intercept

Intercept	-0.28687
Standard error	1.09398
95% lower limit (2-tailed)	-3.32424
95% upper limit (2-tailed)	2.75050
t-value	0.26222
df	4.00000
P-value (1-tailed)	0.40305
P-value (2-tailed)	0.80610

Funnel plot and Egger's regression test for HHS

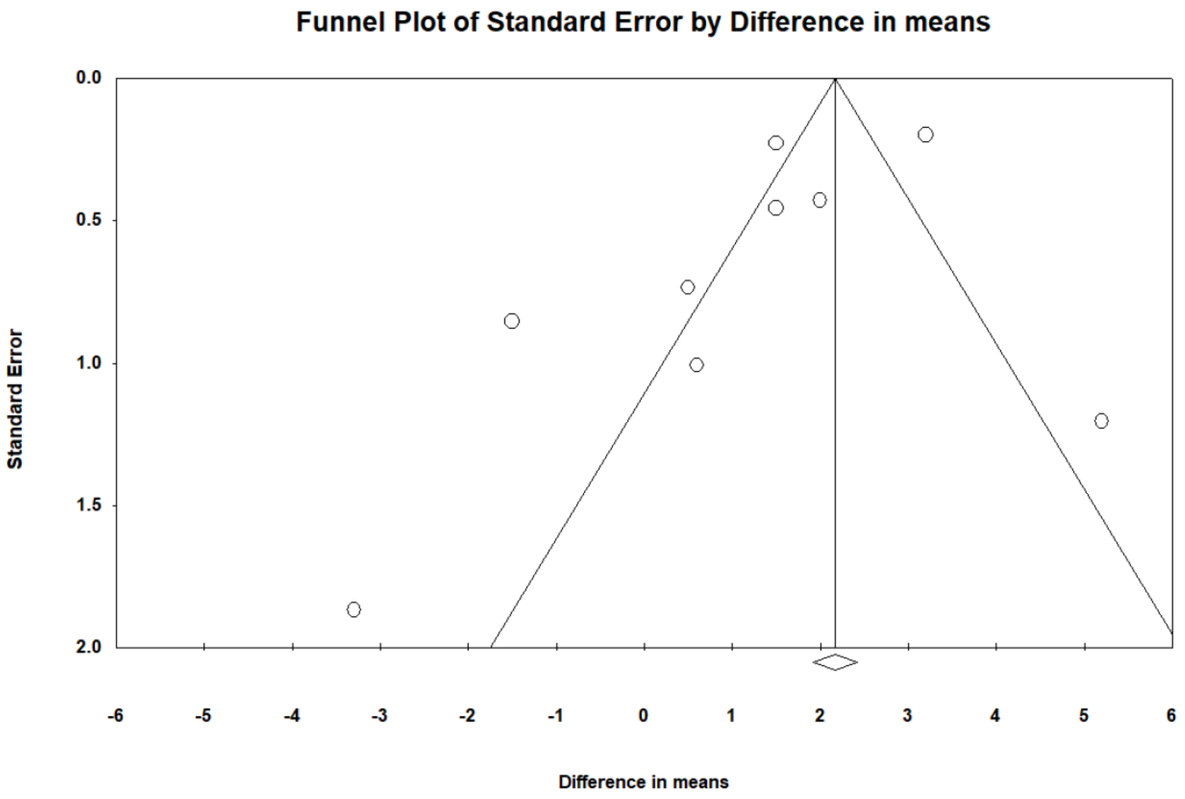

Egger's regression intercept

Intercept	-2.71649
Standard error	1.65051
95% lower limit (2-tailed)	-6.61932
95% upper limit (2-tailed)	1.18634
t-value	1.64585
df	7.00000
P-value (1-tailed)	0.07189
P-value (2-tailed)	0.14379

Funnel plot and Egger's regression test for LLD

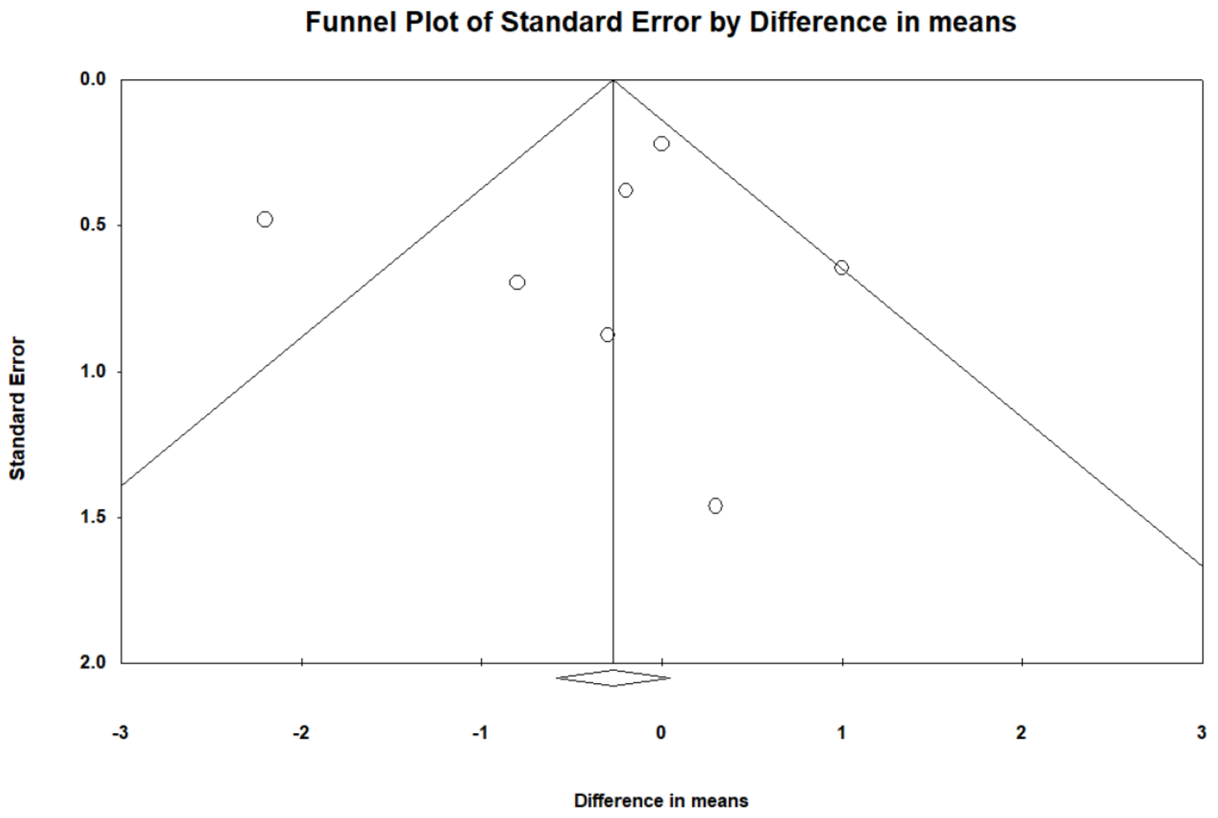

Egger's regression intercept

Intercept	-0.55765
Standard error	1.54611
95% lower limit (2-tailed)	-4.53205
95% upper limit (2-tailed)	3.41676
t-value	0.36068
df	5.00000
P-value (1-tailed)	0.36654
P-value (2-tailed)	0.73308
